# Longitudinal study of changes in greenness exposure, physical activity and sedentary behavior in the ORISCAV-LUX cohort study

**DOI:** 10.1186/s12942-024-00374-7

**Published:** 2024-05-21

**Authors:** Juliette F. E. van Beek, Laurent Malisoux, Olivier Klein, Torsten Bohn, Marion Tharrey, Frank J. Van Lenthe, Mariëlle A. Beenackers, Martin Dijst, Camille Perchoux

**Affiliations:** 1 Department of Urban Development and Mobility, Luxembourg Institute of Socio-Economic Research, 11 Porte Des Sciences, 4366 Esch-Sur-Alzette, Luxembourg; 2 Faculty of Humanities, Education and Social Sciences, Department of Geography and Spatial Planning, University of Luxembourg, 11 Porte Des Sciences, 4366 Esch-Sur-Alzette, Luxembourg; 3 Department of Precision Health, Luxembourg Institute of Health, 1A-B Rue Thomas Edison, 1445 Strassen, Luxembourg; 4 Department of Public Health, Erasmus MC, University Medical Center, Dr. Molewaterplein 40, 3015 GD Rotterdam, The Netherlands; 5 University of Luxembourg, 2 Avenue de L’Universite, 4365 Esch-Sur-Alzette, Luxembourg

**Keywords:** Longitudinal study, Tree cover density, Soil-adjusted vegetation index, Green diversity, Physical activity, Sedentary behavior, Social inequalities

## Abstract

**Background:**

Greenness exposure has been associated with many health benefits, for example through the pathway of providing opportunities for physical activity (PA). Beside the limited body of longitudinal research, most studies overlook to what extent different types of greenness exposures may be associated with varying levels of PA and sedentary behavior (SB). In this study, we investigated associations of greenness characterized by density, diversity and vegetation type with self-reported PA and SB over a 9-year period, using data from the ORISCAV-LUX study (2007–2017, n = 628).

**Methods:**

The International Physical Activity Questionnaire (IPAQ) short form was used to collect PA and SB outcomes. PA was expressed as MET-minutes/week and log-transformed, and SB was expressed as sitting time in minutes/day.

Geographic Information Systems (ArcGIS Pro, ArcMap) were used to collect the following exposure variables: Tree Cover Density (TCD), Soil-adjusted Vegetation Index (SAVI), and Green Land Use Mix (GLUM). The exposure variables were derived from publicly available sources using remote sensing and cartographic resources. Greenness exposure was calculated within 1000m street network buffers around participants’ exact residential address.

**Results:**

Using Random Effects Within-Between (REWB) models, we found evidence of negative within-individual associations of TCD with PA (β = − 2.60, 95% CI − 4.75; − 0.44), and negative between-individual associations of GLUM and PA (β = − 2.02, 95% CI − 3.73; − 0.32). There was no evidence for significant associations between greenness exposure and SB. Significant interaction effects by sex were present for the associations between TCD and both PA and SB. Neighborhood socioeconomic status (NSES) did not modify the effect of greenness exposure on PA and SB in the 1000 m buffer.

**Discussion:**

Our results showed that the relationship between greenness exposure and PA depended on the type of greenness measure used, which stresses the need for the use of more diverse and complementary greenness measures in future research. Tree vegetation and greenness diversity, and changes therein, appeared to relate to PA, with distinct effects among men and women. Replication studies are needed to confirm the relevance of using different greenness measures to understand its’ different associations with PA and SB.

**Supplementary Information:**

The online version contains supplementary material available at 10.1186/s12942-024-00374-7.

## Background

Being sufficiently active is essential for maintaining good health, and physical inactivity contributes majorly to the development of non-communicable diseases (NCD). Worldwide, about 28% of the adult population does not meet the World Health Organization recommendations [1] on physical activity (PA) and is considered physically inactive, with numbers going up to 37% in high-income countries [2]. There is strong evidence that 6–10% of all deaths from NCD can be attributed to physical inactivity [3]. However, research done in the last decades shows it is no longer sufficient to meet minimum PA levels recommended by health guidelines to reduce health risks, by stressing the importance of simultaneously limiting sedentary behavior (SB) [4]. SB includes activities performed in a sitting or reclining position that do not increase energy expenditure levels substantially above the resting level (such as sleeping, sitting and lying down, and engaging in forms of screen-based entertainment) [5].

Physical inactivity and SB are distinctively different and complementary behaviors in the movement expenditure continuum. This imposes a need to address them and their correlates both separately and conjointly to improve our understanding of the mechanisms of both behaviors, and their determinants. Applying a socio-ecological perspective offers opportunities to investigate the determinants of PA and SB on different levels [6–9]. Furthermore, there are potential interactions between the different levels of determinants, and adopting a “systems thinking” approach could be helpful in recognizing the natural complexity of PA and SB and the settings where these behaviors take place [10, 11].

Green spaces are an important environmental characteristic that are linked to health [12, 13], for example through the pathway of providing opportunities for PA [14]. The association between the green environment and SB is far less studied, and evidence for a relationship between the two is to this day, scarce and inconclusive. A study in Denmark showed that SB was more frequent in neighborhoods with less green surroundings [15], while a recent study in Canada found that both PA and SB levels are higher in greener neighborhoods [16].

With urban areas expanding and an increasing amount of people living in urban areas, people tend to experience reduced access to green environments [17, 18]. There are also considerable differences in greenness exposure among geographical areas; greenness in urban areas is rather represented by green alleys, trees alongside the sidewalk [19], or flower pots [20], while in more suburban to rural environments, greenness is rather represented by forests, or green fields. Several studies have shown the importance of street greenery for PA, and specifically cycling as a means of active transport [21, 22]. Importantly however, not all types of green environment will be equally suitable for PA. The diversity of green environments and opportunities for different types of PA that these environments offer, are hardly captured by traditional measures of access and percentage of overall green (e.g. Normalized Difference Vegetation Index (NDVI)) in the neighborhood. There is a need for a more comprehensive understanding if, and how, different types of green environments determine PA and SB. This can be achieved by using measures that account for other aspects of the green environment such as type [17] or diversity of green [24, 25], as the mechanisms between greenness exposure, PA and SB might not be fully captured by a single metric. This is only endorsed by the lack of consensus on the type and amount of greenness exposure needed to maximize health gains in the population [23, 26].

Nowadays, there is growing interest in the relationship between greenness diversity, or in more general terms biodiversity, and health. Biodiversity can be defined as the variability within species (genetic), between species and between ecosystems [27]. As stated previously, it is generally acknowledged that the availability, quality, accessibility and proximity of green spaces determine the magnitude of their positive health effects [28]. However, the association between biodiversity within green spaces and health remains underexplored [24, 29]. Especially in the context of health behaviors, there is a research gap in understanding the pathway between biodiversity, PA and SB. In a review published by Marselle et al. [30], a conceptual pathway is proposed linking (contact with) biodiversity to human health, where diverse green environment facilitate physical activity through its building capacities [30]. Additionally, diverse green environments may potentially be more attractive for recreation and enhancing physical activity levels as it provides sensory stimulation through different pathways, by providing greater psychological restorative benefits [31, 32], and a low stress environment [33]. To address this gap, we included a measure of green land use diversity in this study next to measures of greenness area and type.

Besides, there is evidence that availability and diversity of greenness in urban areas are unequally distributed among sexes [34, 35], social groups and levels of area deprivation [36–38]. On top of that, there is ample evidence for effect modification of levels of disadvantage on the association between green space and health, although there is no clear consensus on the directionality. On one hand, there is evidence that disadvantaged and socially deprived groups benefit less from the positive effects of greenness exposure on health outcomes than people in higher socioeconomic status (SES) groups [38, 39]. On the other hand, the degree of inequality in mortality related to income deprivation tends to be lower for populations with greater greenness exposure than those exposed to less green areas [37]. This is consistent with the results of a recent review on green space and health equity [40]. Additionally, distinct sex differences in the association between green space, PA, and SB can be expected [41, 42], implying both an unequal exposure as an unequal effect of exposure among sexes, social groups and socio-economic areas [43, 44]. However, only few studies investigated if changes in PA and SB due to changes in greenness exposure over time [45, 46] affect these groups differently.

Although there is evidence of an association between greenness exposure, PA and SB from cross-sectional studies, longitudinal studies on the effects of change in greenness exposure and change in PA and SB over time are lacking. Studies that follow the trajectories of individual-level characteristics (such as health, health behaviors, and environmental- and socio-economic determinants) over time are deemed necessary to draw stronger conclusions on causality. Most recent reviews looking into built environmental correlates of PA [47, 48] only identified four longitudinal studies on the association between greenness and PA. Two recent reviews on correlates of SB [49, 50] did not report any longitudinal studies.

This study analyzed longitudinal associations between different types of greenness exposure and PA and SB. We hypothesized that an increase in any type of greenness exposure over time promotes PA and reduces SB. Secondly, it was expected that an increase in green diversity and higher tree density have a stronger association with PA and SB levels over time than overall greenness. Lastly, it was hypothesized that the relationship between (changes in) greenness exposure, PA, and SB differ by sex, and neighborhood SES (NSES). Specifically, we hypothesized that men and residents of deprived neighborhoods benefit less from greenness exposure and diversity, resulting in lower PA and higher SB levels over time.

## Methods

### Study population

**Fig. 1:**
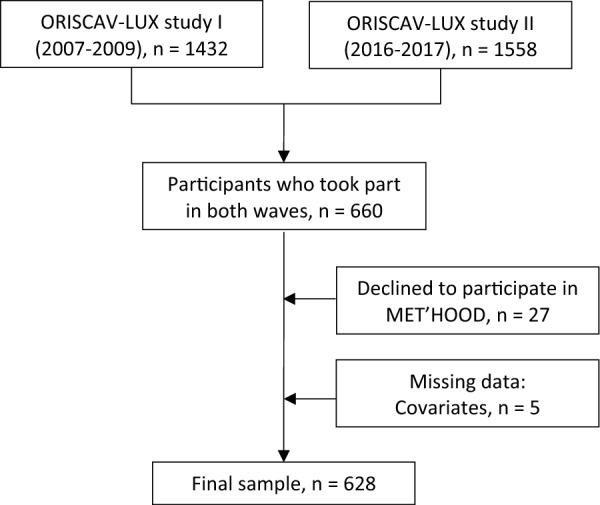
Flow chart of study participant selection

**Fig. 2:**
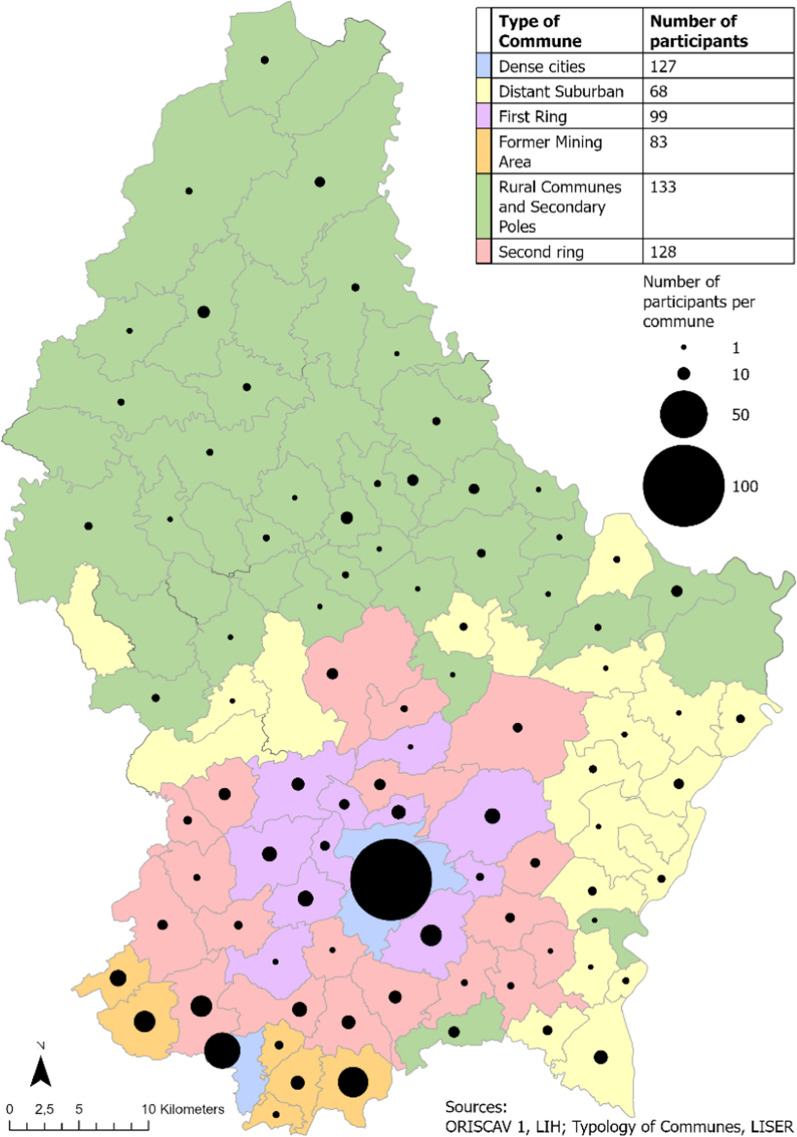
Typology of communes, number of participants per type of commune and aggregated location of participants for study wave 1

This study is based on the ORISCAV-LUX cohort study, a nationwide population-based survey monitoring cardiovascular health in Luxembourg. Details on the ORISCAV-LUX sampling and measurements have been described previously [51, 52]. In short, participants in the first wave (January 2007–2009; n = 1432) were drawn from the National Health Insurance Register (IGSS) by random sampling stratified on age, sex and district, to form a representative sample of the population of Luxembourg. Additional recruitment for the second wave (January 2016–July 2017; n = 1558) was performed to overcome the drop in participants due to refusal to take part in the follow-up studies. Participants completed self-administered questionnaires and attended nurse visits for clinical and anthropometric examinations. In total, 660 participants took part in both waves (46% of baseline sample), of which 27 (2%) did not consent to the use of their data within MET’HOOD. For the present analysis, we additionally excluded participants of which there was still missing data after multiple imputation (n = 5), resulting in a final sample of 628 participants (Fig. 1). The location of the participants, and clustering within geographic areas (i.e. type of commune), is displayed in Fig. 2.

The study was approved by the Luxembourg National Ethics Committee for Research (Ref: 202104/03 V2.0).

### Greenness variables

We considered tree cover density (TCD), soil-adjusted vegetation index (SAVI) and green land use mix (GLUM) as greenness exposure measures (Additional file 1, Image S1), which were derived from open data sources (Table 1) and processed with GIS software (ESRI Inc. ArcMap Version 10.6.1; ArcGIS Pro Version 3.0.3). Main greenness exposure was computed within street network buffers of 1000 m around participants’ exact residential addresses (Additional file 1, Image S2); additional buffers of 500 and 800 m were used for sensitivity analyses. The 1000 m network buffer is commonly used as an exposure measure in PA research, as it corresponds to a walking trip with a reasonable duration of 10-15 min [53–55]. Based on the availability of the data from the open data sources, time points of the collected data range between 2009–2012 for the first wave and 2015–2018 for the second wave.

**Table 1:** Details on data collection points and sources of green- and built environment characteristics

Exposure measure	Time point 1	Time point 2	Spatial resolution (m)	Data	Source
TCD	2012	2018	20 × 20	Copernicus Land Monitoring Service	European Environmental Agency
SAVI	2009	2018	30 × 30	Landsat 7 Enhanced Thematic Mapper (2009) Landsat 8 Operational Land Imager (2018)	U.S. Geological Survey
GLUM	2007	2018		LIS-L Land Use Map	Ministère de l’Environnement, du Climat et du Développement durable & Ministère de l’Énergie et de l’Aménagement du territoire (Le Gouvernement du Grand-Duché de Luxembourg)
Building footprint	2008	2015		Base de Données Topo-Cartographique du Grand-Duché de Luxembourg	Administration du Cadastre et de la Topographie du Grand-Duché de Luxembourg

TCD Tree Cover Density; SAVI Soil-Adjusted Vegetation Index; GLUM Green Land Use Mix

TCD is a satellite-based measure defined as the vertical projection of tree crowns to a horizontal earth’s surface [56]. It is a measure for proportional crown coverage per pixel, ranging from 0 (non-tree covered area) to 1 (total tree cover). The SAVI is also a satellite-based measure, capturing the state of plant health based on the reflection of near-infrared light by plant tissues [57], and corrects for soil reflectance when vegetative cover is low (which is often the case in urban areas). Satellite images of the months of August 2009 and 2018 were used as reference, as they offered the best cloud-free cover. The SAVI is calculated on pixel level using the following formula:

1 $$SAVI = \left( {\left( {NIR - Red} \right)} \right)/\left( {\left( {NIR + Red + L} \right) } \right) \times \left( {1 + L} \right)$$

where: Red = red band reflectance; NIR = near infrared band reflectance; L = soil correction factor (= 0.5).

The final SAVI score is an average index of the density of overall greenness within the network buffer, given in a range of 0–1 with higher values corresponding to higher levels of vegetation density. The GLUM is an indicator for green land use diversity in the network buffer. We selected all land use classes that can be considered as green (n = 17), namely: parks, golf courses, campgrounds, arable land, grassland, special agriculture (wine, fruit trees, orchard, other), forest (coniferous, mixed, deciduous, young), natural grassland, heathland, bushes, and wetland. For each participant, we calculated the land cover of each land use class as a percentage of the total buffer area. Based on these percentages, an entropy score was calculated using the following equation:

2 $$GLUM = \left( { - \sum_{k} \left( {p_{k} *{\text{ln}}p_{k} } \right) } \right)/{\text{ln}}T_{k}$$

where: p_k_ = percent of land use k within the network buffer; T_k_ = total number of land use classes.

The GLUM ranges from 0 to 1, with higher scores indicating a more equal distribution of the 17 different land use classes in the network buffer. A correlation matrix (Spearman) between the three greenness measures is included in Additional file 1 (Table S1).

### Assessment of PA and SB

PA and SB outcomes were assessed at both waves using the International Physical Activity Questionnaire (IPAQ) short form [58]. Participants estimated their PA over the last seven days, by reporting the number of days where they practiced vigorous, moderate, and walking activity, respectively, as well as the amount of time that was usually spent on these activities. From the answers, we calculated the metabolic equivalents per minute (MET-min) per week for each activity category and summed it into a single measure for PA. For SB, the IPAQ included the following question: ‘*During the last 7 days, how much time did you spend sitting on a week day?*’ where participants stated the time usually spent sitting on any day.

### Covariates

Included time-invariant covariates were biological sex and relocation status during the study period (non-mover, mover). Time-variant covariates were age, marital status (married/living with partner, single, divorced/separated/widowed), educational level (no diploma, secondary diploma, university diploma).

Lifestyle preference [for being active] (little to no importance, important) is included as time-invariant covariate, as we hypothesized that a preference is rather stable over time compared to the actual behavior. A chi-squared test confirmed that lifestyle preference [for being active] did not significantly change over the study period. As season is related to greenness levels throughout the year and seasonal differences in PA levels [59–61], the date of questionnaire completion was added as time-varying covariate. The average housing price (in euros per m^2^) in the municipality of residence was used as measure of NSES [62–65] and added as time-variant covariate. Area deprivation can be linked to lower PA levels [66], and the quality of and perceived access to green spaces [67, 68]. Degree of urbanicity was determined by taking the building area surface (i.e. footprint) of residential and non-residential buildings [69], calculated as percentage of the total buffer area. The distinction between residential and non-residential building density accounts for possible diverging pathways in their relationships with PA and SB. Both were considered time-variant. Urbanicity is directly related to the amount of green space available, and is known to influence the pathway between greenness exposure and health outcomes [70]. Spearman correlations between residential-, non-residential building density and housing price with the greenness exposure measures are reported in Additional File 1, Table S1.

### Statistical analysis

Multiple imputation (m = 60) was performed on the whole ORISCAV-LUX cohort (1432 respondents in Wave 1, 1558 respondents in Wave 2) using the MICE (Multiple Imputation by Chained Equations) algorithm [71] to deal with missing outcome data from the self-reported IPAQ questionnaire (See Additional file 1 for a description of the imputation method). Complete case analysis of the current study sample would have resulted in the loss of 270 participants (43% of study sample). Multiple imputation made it possible to retain 628 out of the eligible 633 participants.

We used a linear REWB model to assess the longitudinal associations between the three different greenness measures and PA and SB outcomes over 9 years. The model included two levels accounting for repeated observations across participants. We ran models for each exposure measure (TCD, SAVI and GLUM) with PA and SB separately. Normality tests showed that the PA data was non-normally distributed and therefore log-transformed. For interpretation purposes, the log-transformed coefficient is exponentiated, which gives the multiplicative factor for every one-unit increase in the independent variable. This means that for every one-unit increase in the independent variable (i.e. the greenness exposure measure), the dependent variable (either PA or SB) increases or decreases by the factor of the exponentiated coefficient [72].

All models included a random effect for each individual participant. The REWB model uses a mean centering approach, and decomposes the time-varying exposure variables (TCD, SAVI and GLUM) and covariates into a between-individual and within-individual component. The between-individual component relates to how exposure across all participants affects the outcome, and the within-individual component represents how change in the exposure relates to changes in the outcome for each individual. All analysis were performed using the lmer function of the lme4 package in R [73], using the ‘simple’ REWB model [74, 75]. The main model was specified as following:

3 $$y_{it} = \beta_{0} + \beta_{1W} \left( {x_{it} - \overline{x}_{i} } \right) + \beta_{2B} \overline{x}_{i} + z^{\prime}_{i} \alpha + \gamma_{it} \delta + \left( {v_{i} + \varepsilon_{it} } \right)$$

where y_it_ indicates the outcome (either PA or SB) for individual i at time t, and x_it_ the time-varying greenness exposure variable (TCD, SAVI or GLUM). The exposure variable x_it_ is decomposed into two parts: a within-individual component (β_1W_) representing the individual’s average effect, and a between-individual (β_2B_) component. The effects of time invariants covariates (z_i_) are represented by vector $\alpha$, and vector $\delta$ represents the effects of time-varying covariates $\gamma$_it_. v_i_ is the model’s random effect for individuals i, and $\varepsilon$_it_ are the model’s level-1 residuals. Interaction terms of greenness exposure (both the between- and within-individual component) with sex (model 2) and NSES (model 3) were investigated in separate models. To explore potential self-selection bias, interactions between exposure and lifestyle preference are tested (model 4). Participants that prefer an active lifestyle might choose to live in greener environments to facilitate this preference. Interaction terms were assessed with a cut-off p-value of 0.1 to raise the Type 1 error rate [76, 77]. For statistically significant interactions, outcomes by group were predicted using linear regression prediction. Change in NSES was categorized in two groups by median change in housing price over the 9-year study period (no to small change: change in housing price < 1222 €/m^2^, increase: change in housing price ≥ 1222 €/m^2^). To check consistency of the results among movers and non-movers, we explored the interaction term of both components of greenness exposure (between- and within-individual) with relocation status. The models used for the sensitivity analyses include interaction terms of the covariate of interest with the between- and within-individual components, and were specified as following:

for time varying covariates (i.e. neighborhood SES)

4 $$y_{it} = \beta_{0} + \beta_{1W} \left( {x_{it} - \overline{x}_{i} } \right) + \beta_{2B} \overline{x}_{i} + \beta_{3} Z_{i} + \beta_{4} \gamma_{i} + \beta_{5W} \left( {x_{it} - \overline{x}_{i} } \right) \times \gamma_{it} + \beta_{6B} \overline{x}_{i} \times \gamma_{it} + \left( {v_{0i} + v_{1i} x_{it} + \varepsilon_{it} } \right)$$

for time invariant covariates (i.e. sex, relocation status)

5 $$y_{it} = \beta_{0} + \beta_{1W} \left( {x_{it} - \overline{x}_{i} } \right) + \beta_{2B} \overline{x}_{i} + \beta_{3} z_{i} + \beta_{4} \gamma_{i} + \beta_{5W} \left( {x_{it} - \overline{x}_{i} } \right) \times z_{i} + \beta_{6B} \overline{x}_{i} \times z_{i} + \left( {v_{i} + \varepsilon_{it} } \right)$$

Sensitivity analyses were performed on smaller definitions of the residential neighborhood to check the robustness of the associations, by running separate models for each greenness exposure in the 500 and 800 m street network buffers.

## Results

### Description of individual and environmental characteristics

Of the 628 participants, the average age at baseline was 44 (± 12) years and 49% were females. Lifestyle preference [for being active] did not change for the majority of the participants (81.53%). The socio-demographics of the current population were similar to the participants of ORISCAV-LUX, with the exception that the current study sample tended to be more educated (Additional file 2, Table S1).

In total, 32% of the participants relocated between the two study waves. Significant changes were witnessed in non-residential building density (mean difference 3%), but not in residential building density (mean difference − 1%) (Table 2). Description of characteristics by sex, NSES, lifestyle preference, and relocation status are displayed in Additional file 2, Tables S2–S5. Participants were exposed to an average TCD of 16% in wave 1, which decreased to 12% in wave 2 (Table 2). The SAVI decreased from 0.41 to 0.36 between the two study waves and the GLUM did not significantly change (both waves 0.31). For a description of greenness in all buffer sizes, see Additional file 2, Table S6.

**Table 2:** Description of participants’ individual and environmental characteristics based on the original dataset (n = 628)

Variables	Mean ± SD or proportion (%)						
	Wave 1			Wave 2			*Test statistics*
	n		*# missing*	n		*# missing*	
Individual-level characteristics							
Age	628	44.05 ± 11.97	0	628	51.96 ± 11.88	0	p < 0.001^1^
Sex	628		0	628		0	
Women	309	48.97		309	48.97		
Men	319	51.02		319	51.02		
Marital status	628		0	628		0	p < .01^2^
Married / Living with partner	468	74.41		487	77.25		
Single	100	16.11		62	9.95		
Divorced/Separated/Widowed	60	9.48		79	12.72		
Education	624		4	623		5	p = .02^2^
No diploma	107	17.01		81	13.00		
Secondary level	305	49.28		290	46.55		
University level	212	33.70		252	40.45		
Lifestyle preference—Importance of PA	628		0	628		0	p = .19^2^
Important	518	82.48		536	85.35		
Little to no importance	110	17.52		92	14.65		
Season (questionnaire completion date)	628		0	628		0	p < .001^2^
Spring (March, April, May)	171	27.23		180	28.66		
Summer (June, July, August)	103	16.40		184	29.30		
Autumn (September, October, November)	150	23.89		111	17.68		
Winter (December, January, February)	204	32.48		153	24.36		
Environmental-level characteristics							
Relocation status	628		0	628		0	
Non mover	433	68.95		433	68.95		
Relocated	195	31.05		195	31.05		
Average housing price in the municipality (€/m^2^)	628	3456 (3221–4107)	0	628	4624 (4298–5715)	0	p < .001^3^
Building density							
Residential buildings		0.07 ± 0.05	0	628	0.07 ± 0.05	0	p = 0.78^1^
Non-residential buildings	628	0.02 ± 0.02	0	628	0.03 ± 0.02	0	p < 0.001^1^
Exposure measures							
TCD	628	0.17 ± 0.11	0	628	0.13 ± 0.11	0	p < 0.001^1^
SAVI	628	0.42 ± 0.09	0	628	0.37 ± 0.06	0	p < 0.001^1^
GLUM	628	0.31 ± 0.10	0	628	0.31 ± 0.10	0	p = 0.44^1^

^1^ Paired t-test for normally distributed continuous variables, ^2^chi-squared test for categorical variables, ^3^Wilcoxon-signed rank test for non-normally distributed variables. Tree cover density (TCD) is given as percentage of the buffer surface, ranging from 0–1; Soil adjusted vegetation index (SAVI) is given as the average SAVI score within the buffer, ranging from 0–1; Green land use mix (GLUM) represents an entropy score, given in a range from 0–1, with higher scores indicating a more equal mix of all green land use types within the buffer

Table 3 gives median values and interquartile range (IQR), and the number of missing data for the PA and SB outcomes of the original data, before multiple imputation. There was no evidence that participants changed their PA levels over the study period, but participants decreased their SB on average by about 30 min.

**Table 3:** Description of outcome measures in the original data (n = 628)

	Wave 1			Wave 2			Test statistics^1^
	n	Median (IQR)	# missing	n	Median (IQR)	# missing	P-value
Original data							
MET-minutes/week	598	3741 (1759–6309)	30	381	3492 (1432–6880)	247	0.60
Sitting time (minutes/day)	623	360 (240–540)	5	503	330 (210–480)	125	** < 0.001**

^**1**^ Wilcoxon signed-rank test

### Main analyses

#### Greenness and PA

REWB models provided evidence for a negative within-individual association of TCD and PA (β = − 2.60, 95% CI − 4.75; − 0.44, Table 4) and a negative between-individual association of GLUM on PA (β = − 2.02, 95% CI − 3.73; − 0.32, Table 4). This indicates that a one-unit increase in TCD and SAVI are associated with a decrease by a factor of respectively exp (− 2.60) = 0.07 and exp (− 2.02) = 0.13 in PA. SAVI was not significantly associated with PA for either the within- and the between-component (Table 4).

**Table 4:** REWB model associations between greenness exposure measures and PA in the 1000 m buffer

	Model 1					Model 2					Model 3					Model 4				
	β	Exp(β)	95% CI		P	β	Exp(β)	95% CI		P	β	Exp(β)	95% CI		P	β	Exp(β)	95% CI		P
TCD																				
Between effect	− 0,44	0,65	− 1,49	0,62	0,41	− 1,36	0,26	− 2,90	0,18	0,08	− 0,74	0,48	− 6,02	4,54	0,78	− 0,22	0,80	− 1,36	0,91	0,70
Female						− 0,18	0,84	− 0,54	0,18	0,33										
TCD*Female						**1,75**	**5,74**	− **0,24**	**3,74**	**0,09**										
Housing price											0,00	1,00	0,00	0,00	0,45					
TCD*Housing price											0,00	1,00	0,00	0,00	0,91					
Lifestyle preference																− 0,43	0,65	− 0,90	0,05	0,08
TCD* Lifestyle preference																− 1,12	0,33	− 3,62	1,38	0,38
Within effect	− **2,60**	**0,07**	− **4,75**	− **0,44**	**0,02**	− **4,31**	**0,01**	− **7,07**	− **1,56**	**0,00**	− **2,50**	**0,08**	− **4,69**	− **0,30**	**0,03**	− **2,63**	**0,07**	− **4,99**	− **0,26**	**0,03**
Female						− 0,18	0,84	− 0,54	0,18	0,33										
TCD*Female						**4,14**	**63,08**	**0,16**	**8,13**	**0,04**										
Housing price change											0,00	1,00	0,00	0,00	0,67					
TCD*Housing price change											0,00	1,00	0,00	0,00	0,50					
Lifestyle preference																− 0,43	0,65	− 0,90	0,05	0,08
TCD* Lifestyle preference																− 0,02	0,98	− 5,42	5,37	0,99
SAVI																				
Between effect	− 1,38	0,25	− 4,76	2,00	0,42	− 0,48	0,62	− 4,35	3,39	0,81	− 3,69	0,03	− 11,78	4,41	0,37	− 1,27	0,28	− 4,64	2,10	0,46
Female						0,80	2,23	− 0,49	2,09	0,22										
SAVI*Female						− 1,82	0,16	− 5,03	1,38	0,26										
Housing price											0,00	1,00	0,00	0,00	0,35					
SAVI*Housing price											0,00	1,00	0,00	0,00	0,51					
Lifestyle preference																− 0,26	0,77	− 2,06	1,54	0,78
TCD* Lifestyle preference																− 0,77	0,46	− 5,25	3,72	0,74
Within effect	0,16	1,17	− 3,69	4,00	0,94	− 0,85	0,43	− 5,33	3,63	0,71	0,22	1,24	− 3,65	4,09	0,91	0,75	2,11	− 3,30	4,80	0,72
Female						0,80	2,23	− 0,49	2,09	0,22										
SAVI*Female						2,07	7,95	− 2,40	6,55	0,36										
Housing price change											0,00	1,00	0,00	0,00	0,67					
SAVI*Housing price change											0,00	1,00	0,00	0,00	0,68					
Lifestyle preference																-0,26	0,77	-2,06	1,54	0,78
TCD* Lifestyle preference																-3,20	0,04	-9,44	3,05	0,32
GLUM																				
Between effect	− **2,02**	**0,13**	− **3,73**	− **0,32**	**0.02**	− 1,47	0,23	− 3,43	0,50	0,14	− 3,08	0,05	− 8,57	2,41	0,27	− **2,23**	**0,11**	− **3,99**	− **0,48**	**0,01**
Female						0,48	1,62	− 0,21	1,18	0,17										
GLUM*Female						− 1,27	0,28	− 3,34	0,80	0,37										
Housing price											0,00	1,00	0,00	0,00	0,41					
GLUM*Housing price											0,00	1,00	0,00	0,00	0,68					
Lifestyle preference																− **1,04**	**0,35**	− **1,99**	− **0,10**	**0,03**
TCD* Lifestyle preference																1,40	4,04	− 1,33	4,12	0,31
Within effect	− 0,18	0,84	− 4,02	3,66	0,93	0,87	2,39	− 3,63	5,37	0,70	− 0,02	0,98	− 3,90	3,85	0,99	0,40	1,49	− 3,48	4,27	0,84
Female						0,48	1,62	− 0,21	1,18	0,17										
GLUM*Female						− 2,58	0,08	− 8,19	3,02	0,23										
Housing price change											0,00	1,00	0,00	0,00	0,68					
GLUM*Housing price change											0,00	1,00	0,00	0,01	0,32					
Lifestyle preference																− **1,04**	**0,35**	− **1,99**	− **0,10**	**0,03**
TCD* Lifestyle preference																− 6,77	0,00	− 15,72	2,18	0,14

Log-transformed coefficients are displayed. PA is expressed as MET-minutes per week. Between- and within-effects refer to the between- and within-component of the exposure measure and were assessed within the same model. We ran separate models for each exposure measure, i.e. *TCD* Tree Cover Density; *SAVI* Soil-Adjusted Vegetation Index; *GLUM* Green Land Use Mix. Significant results are displayed in bold

Model 1 adjusted for: sex, lifestyle preference, age, education, marital status, housing price, nonresidential and residential building density, date of questionnaire completion, relocation status; Model 2 adjusted for: sex, lifestyle preference, age, education, marital status, housing price, nonresidential and residential building density, date of questionnaire completion, relocation status, and the interaction terms of the between- and within-individual components of the exposure measure with sex; Model 3 adjusted for: sex, lifestyle preference, age, education, marital status, housing price, nonresidential and residential building density, date of questionnaire completion, relocation status, and the interaction terms of the between- and within-individual components of the exposure measure with the between- and within-individual components housing price; Model 4 adjusted for: sex, lifestyle preference, age, education, marital status, housing price, nonresidential and residential building density, date of questionnaire completion, relocation status, and the interaction terms of the between- and within-individual components of the exposure measure with lifestyle preference [for being active]

**Fig. 3:**
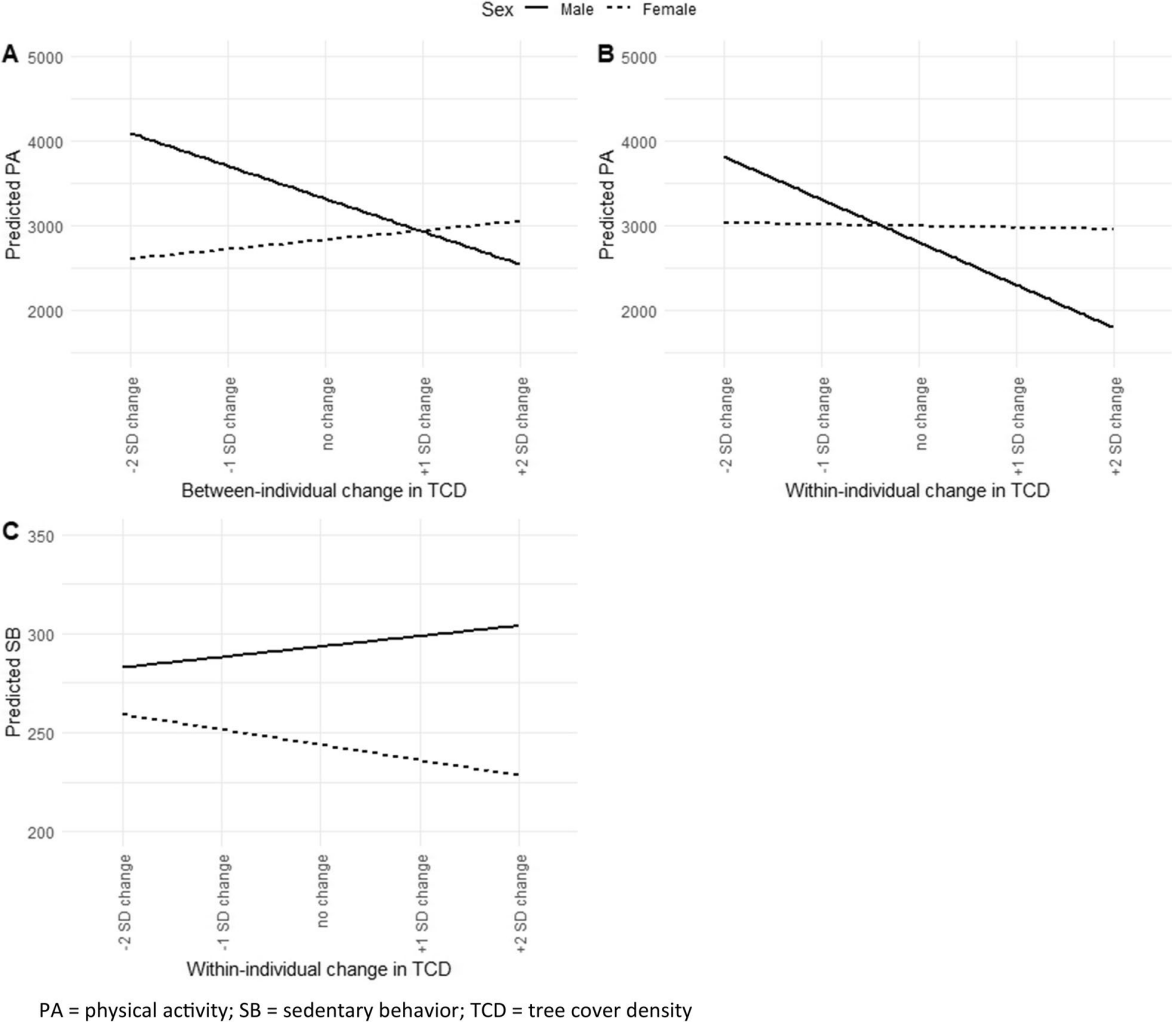
Results from regression prediction by sex. PA physical activity; SB sedentary behavior; TCD tree cover density

Significant interactions by sex indicated that the association of the between-individual component of TCD and PA differed between men and women (p = 0.09), as well as the within-component of TCD and PA (p = 0.04) (both Table 4). The latter represents different responses to changes in TCD and PA on the individual-level by sex. Figure 3 shows that an increase in TCD was associated with a decrease in PA for men, and an increase in PA for women (Fig. 3A), and that a within-individual increase in TCD leads to a decrease in PA only among men (Fig. 3B).

No significant interaction effects were observed for any of the greenness exposure indicators, NSES and lifestyle preference [for being active] within the 1000-m buffer (Table 4).

#### Greenness and SB

No associations between TCD, SAVI, GLUM and SB were observed (Table 5). Significant interactions by sex indicated that the association of the within-individual component of TCD and SB differed between men and women (p = 0.03) (Table 5). The regression plot shows that men become more and women become less sedentary with an increase in TCD over time (Fig. 3C).

**Table 5:** REWB model associations between greenness exposure measures and SB in the 1000 m buffer

	Model 1				Model 2				Model 3					Model 4			
	β	95% CI		P	β	95% CI		P	β	95% CI			P	β	95% CI		P
Sedentary behavior																	
TCD																	
Between effect	− 0,43	− 125,50	124,64	0,99	− 93,71	− 270,46	83,04	0,30	172,25		− 491,00	835,51	0,61	14,42	− 120,62	149,45	0,83
Female					− **77,54**	− **120,89**	− **34,19**	**0,00**									
TCD*Female					176,16	− 65,55	417,87	0,15									
Housing price									0,02		− 0,01	0,05	0,16				
TCD*Housing price									− 0,04		− 0,19	0,11	0,60				
Lifestyle preference														40,25	− 12,32	92,83	0,13
TCD*Lifestyle preference														− 67,52	− 350,01	214,98	0,64
Within effect	− 50,11	− 270,13	169,91	0,65	122,36	− 151,45	396,18	0,38	− 50,93		− 270,44	168,57	0,65	− 90,94	− 331,87	149,98	0,46
Female					− **77,54**	− **120,89**	− **34,19**	**0,00**									
TCD*Female					− **418,95**	− **808,24**	− **29,65**	**0,03**									
Housing price change									0,01		− 0,01	0,04	0,22				
TCD*Housing price change									0,09		− 0,23	0,40	0,59				
Lifestyle preference														40,25	− 12,32	92,83	0,13
TCD*Lifestyle preference														205,00	− 378,86	788,86	0,49
SAVI																	
Between effect	205,15	− 214,56	624,85	0,34	249,70	− 203,95	703,34	0,28	− 86,82		− 1025,86	852,22	0,86	279,12	− 145,06	703,29	0,20
Female					− 14,63	− 156,27	127,01	0,84									
SAVI*Female					− 91,11	− 446,74	264,52	0,62									
Housing price									− 0,01		− 0,09	0,07	0,78				
SAVI*Housing price									0,06		− 0,15	0,27	0,55				
Lifestyle preference														**214,64**	**34,55**	**394,73**	**0,02**
SAVI*Lifestyle preference														− **462,00**	− **902,93**	− **21,07**	**0,04**
Within effect	10,82	− 348,77	370,41	0,95	71,98	− 342,36	486,32	0,73	12,19		− 347,18	371,55	0,95	3,19	− 370,92	377,30	0,99
Female					− 14,63	− 156,27	127,01	0,84									
SAVI*Female					− 123,39	− 540,28	293,51	0,56									
Housing price change									0,01		− 0,01	0,04	0,24				
SAVI*Housing price change									− 0,25		− 0,67	0,16	0,23				
Lifestyle preference														**214,64**	**34,55**	**394,73**	**0,02**
SAVI*lifestyle preference														130,12	− 497,52	757,76	0,68
GLUM																	
Between effect	183,14	− 29,08	395,37	0,09	229,21	− 8,63	467,05	0,06	23,17		− 647,86	694,20	0,95	217,18	− 0,53	434,90	0,05
Female					− 17,24	− 97,79	63,30	0,67									
GLUM*Female					− 104,83	− 350,80	141,15	0,40									
Housing price									0,00		− 0,05	0,05	0,92				
GLUM*Housing price									0,04		-0,12	0,20	0,62				
Lifestyle preference														91,15	− 12,07	194,37	0,08
GLUM*Lifestyle preference														− 189,86	− 486,98	107,27	0,21
Within effect	− 6,65	− 406,19	392,89	0,97	− 7,41	− 465,01	450,20	0,97	− 5,79		− 405,66	394,07	0,98	38,79	− 364,11	441,69	0,85
Female					− 17,24	− 97,79	63,30	0,67									
GLUM*Female					2,46	− 521,84	526,76	0,99									
Housing price change									0,01		-0,01	0,04	0,22				
GLUM*Housing price change									-0,07		-0,48	0,34	0,74				
Lifestyle preference														91,15	− 12,07	194,37	0,08
GLUM*Lifestyle preference														− 478,80	− 1387,45	429,86	0,30

SB is expressed as sitting time in minutes per day. Between- and within-effects refer to the between- and within-component of the exposure measure and were assessed within the same model. We ran separate models for each exposure measure, i.e. *TCD* Tree Cover Density; *SAVI* Soil-Adjusted Vegetation Index; *GLUM* Green Land Use Mix. Significant results are displayed in bold

Model 1 adjusted for: sex, lifestyle preference, age, education, marital status, housing price, nonresidential and residential building density, date of questionnaire completion, relocation status; Model 2 adjusted for: sex, lifestyle preference, age, education, marital status, housing price, nonresidential and residential building density, date of questionnaire completion, relocation status, and the interaction terms of the between- and within-individual components of the exposure measure with sex; Model 3 adjusted for: sex, lifestyle preference, age, education, marital status, housing price, nonresidential and residential building density, date of questionnaire completion, relocation status, and the interaction terms of the between- and within-individual components of the exposure measure with the between- and within-individual components housing price; Model 4 adjusted for: sex, lifestyle preference, age, education, marital status, housing price, nonresidential and residential building density, date of questionnaire completion, relocation status, and the interaction terms of the between- and within-individual components of the exposure measure with lifestyle preference [for being active]

**Fig. 4:**
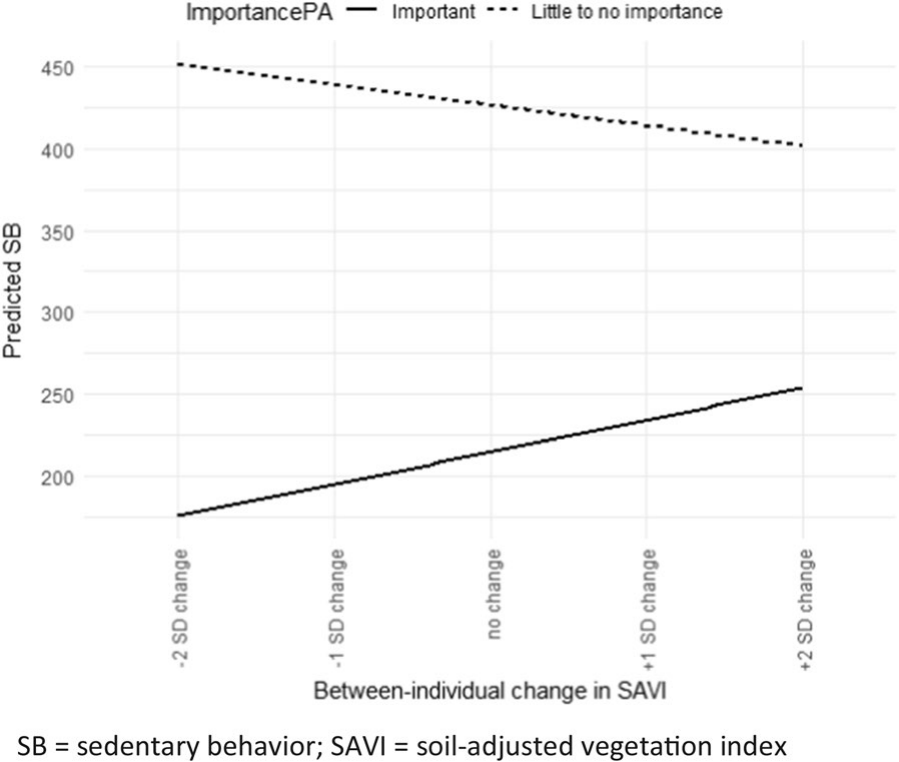
Results for regression prediction by lifestyle preference [for being active]. SB sedentary behavior; SAVI soil-adjusted vegetation index

For SB, no significant interaction effects were observed for any of the greenness exposure measures and NSES within the 1000-m buffer. However, there was evidence for a significant interaction between the between-individual component of SAVI and lifestyle preference [for being active] (p = 0.04) (both Table 5). The regression plot shows that participants with a preference for being active display higher SB levels with increasing levels of SAVI, while participants that do not have this preference tend to show the opposite (Fig. 4).

### Sensitivity analyses

#### Greenness and PA

Results of the sensitivity analyses using 500 and 800 m buffer sizes are presented in Additional file 3. Significance of the negative association of within-individual change in TCD and PA, and the negative between-individual association of GLUM and PA was consistent across the different buffer sizes. Effect modification of sex and NSES were explored in 500m and 800m buffers (Additional file 3, Tables S1–S4). The interaction of the between-individual component of TCD and sex on PA disappeared in the smaller buffer sizes. The observed interaction of sex and within-individual change in TCD on PA is consistent in the 800 m buffer, but not the 500 m buffer. In the 500m buffer, none of the interactions observed reached significance. We did not observe effect modification of NSES on the associations between TCD, SAVI, GLUM and PA.

Results of the sensitivity analyses on lifestyle preference [for being active] and relocation status are presented in Additional file 3, Tables S5–S10. For lifestyle preference [for being active], a significant interaction with the within-individual component of GLUM showed up in the 500 m buffer (p < 0.01), indicating a decrease in PA with increasing levels of GLUM for participants that give little to no importance to being active (Additional file 3, Table S5, Fig S3A). No significant interactions with relocation status were observed in the 1000m buffer. Sensitivity analyses on the smaller buffer sizes showed that the results for relocation status are not consistent with the 1000 m buffer (Additional file 3, Table S9). We observed a significant interaction of the between-individual component of TCD and relocation status on PA in the 500m buffer (p = 0.06), with participants that relocated demonstrating a stronger decrease in PA with increasing TCD (Additional file 3, Fig S5C).

#### Greenness and SB

Similar to the main analysis, no significant effects of TCD, SAVI and GLUM with SB were observed for the 500m and 800m buffers. The observed interaction of sex and within-individual change in TCD and SB was consistent in the 800 m buffer (Additional file 3, Figure S1), but not the 500 m buffer. For NSES, we observed a significant interaction for within-individual change in SAVI and NSES on SB only in the 500m buffer (p = 0.08). For participants that experienced no to a small change in NSES, a change in SAVI has a smaller positive effect on SB than for participants who experience a strong increase in NSES (Additional file 3, Figure S2).

Additionally, the interaction between the between-individual component of SAVI and lifestyle preference [for being active] was consistent in the 500 and 800 m buffer (Additional file 3, Table S16, Figures S3B-S4). For relocation status, significant interactions with the between-individual component of SAVI in both the 500 m (p = 0.04) and 800m (p = 0.06) buffer, and an interaction with the between-individual component of GLUM (p = 0.07) in the 500 m buffer were observed (Additional file 3, Table S10). Participants that relocated are more negatively impacted by changes in greenness, by demonstrating a stronger increase in SB with increasing SAVI, and a stronger increase in SB with increasing GLUM (Additional file 3, Figures S5A, S5B and S6).

## Discussion

This study provides new insights on longitudinal associations between greenness exposure, PA and SB, and the varying effects between types of greenness measures used. Surprisingly, we found that an individual-level increase in TCD was significantly associated with a decrease in PA. Additionally, a significant between-individual association in GLUM indicated that being exposed to higher levels of GLUM was associated with lower PA. None of the main associations were significant for SB (hypothesis 1). These results confirm our second hypothesis, stating that measures of tree vegetation and greenness diversity are more strongly associated with PA levels (hypothesis 2). Furthermore, our results provide evidence for effect modification by sex for exposure to TCD on PA and SB, with men displaying lower levels of PA and higher levels of SB with increasing TCD compared to women. Additionally, the negative association between within-individual change in SAVI in the 500m buffer and SB was significantly modified by changes in area deprivation, indicating that participants living in deprived areas decrease their SB less with increasing overall greenness (hypothesis 3). There was a consistent significant interaction between the between-individual component of SAVI and lifestyle preference [for being active] in association with SB, which could indicate that participants with a preference for an active lifestyle do not benefit from increasing levels of greenness regarding their SB levels.

Most studies assessing the association of greenness exposure with PA and SB are cross-sectional. The majority of these studies report positive associations between greenness and PA [78–82] and SB [15, 82], although some studies report no significant associations [83–85]. Longitudinal studies investigating the association between greenness exposure and PA and SB are scarce, and results are rather inconsistent. Results range from no effect of greenness exposure to PA outcomes [86] to positive effects [16, 87]. These inconsistencies are most likely the result of differences in study design, such as differences in the study population, greenness and outcome measures used, duration of study period, and used statistical methods [88]. A recent review article by Cardinali et al. [23] addressed these inconsistencies and proposed guiding principles to enhance synchronization of studies in the field of green space and health.

The results of the current study, stating that PA levels decrease with an increase in exposure to TCD and GLUM, are inconsistent with the current literature. However, our results could be partly explained by sex differences. Although it is common to adjust [85–87] or stratify models by sex [16, 39], interactions by sex are rarely included. The advantage of including an interaction by sex over stratification is that we can test the significance of the interaction by sex and estimate the effect of sex on the outcome, which is not possible using stratification. We found that the significant association between TCD and PA was mainly driven by a negative association for men, with men displaying lower levels of PA on both the group- as individual-level with increasing exposure to TCD. Although negative, the stronger association of exposure to TCD among men is conflicting with recent findings that women generally tend to show a stronger relationship between greenness exposure and health [42].

A previous study using the ORISCAV-LUX cohort actually showed that the proportion of highly active participants increased more for men than for women over the study period [89]. As the decrease in TCD over the study period is limited (-4%), the negative association between TCD and PA in men could be driven by unmeasured confounders such as change in other built environment characteristics over time. Indeed, urban green spaces are eminently suited for lower intensity activities such as walking, jogging, or group activities such as yoga [90], which have a strong social setting [91] and might be more attractive to the female population. Men tend to be more motivated to participate in sports by elements of competition [92], which rather take place in organized settings, such as club-organized sports. Availability of sport courts, indoor gym equipment and participation in organized sports clubs are shown to be associated with higher levels of vigorous PA (VPA) [93, 94], but null associations are also witnessed [95]. However, this could be due to a discrepancy in objective and perceived availability, and typology of recreational facilities [96, 97]. There might have been a change in the availability and/or diversity facilities for sport and physical activity in the neighborhoods were participants lived, which could have contributed to higher increases of VPA among men.

Although we did not find any longitudinal associations between the SAVI and PA and SB, we observed a significant modification effect of NSES on the association of SAVI and SB in the 500m buffer. These results indicated that participants living in deprived areas decrease their SB levels less than participants in more affluent areas with increasing greenness do. As the SAVI captures all available vegetation, it might better describe greenness exposure in smaller buffer sizes compared to TCD or GLUM. Trees and land use classes considered as being green are probably less available in the immediate residential environment compared to an overall greenness measures. Neighborhood deprivation likely influences the quality and maintenance of green areas in the neighborhood, and hence their supportive effect on PA behaviors. The effects of urban vegetation on PA have been shown to vary among demographic groups and NSES [98]. For example, NSES moderated the association between park safety and PA in Hong Kong [99], indicating that park safety significantly affected park-based PA only for those living in low-income neighborhoods.

We witnessed a significant negative between-individual association between GLUM and PA, and an almost significant association with SB, indicating that an increase in GLUM leads to a decrease in PA and an increase in SB. Unsuitability of certain green land use classes for PA could perhaps explain these negative associations, in a way that exposure to a diverse green environment does not necessarily imply a pleasant or functional context for recreational purposes and exercise [82]. Urban green has been linked to more sports participation and bicycling, and agricultural green with more gardening and odd jobs [85]. Swampy areas, dense shrub layers and deadwood could cause negative perceptions of nature, and half-open areas with mown lawns, scattered trees and shrubs are perceived as more pleasant [100]. This suggests a presence of competing interests of green land use classes, where the presence of a land use class unsuitable for PA might diminish the effects of a supportive land use class. An important aspect to consider is that we did not take accessibility of green land use classes, in terms of public or private ownership, into account.

## Strengths and limitations

Main strengths of this study include its longitudinal design and the assessment of the between- and within-individual effects of different types of greenness exposure measures using a hybrid model. We assessed greenness exposure and PA and SB levels over a relatively long follow-up (9 year) on a countrywide scale, which is a sufficient time-period and scale for environmental changes and variation to occur. Additionally, we used objectively measured environmental variables. Sensitivity analyses on buffer sizes emphasized the robustness of the main associations, but associations decreased in strength in smaller definitions of the neighborhood. Smaller buffer sizes seemed to play a bigger role in effect modification of NSES, and the sensitivity analysis on relocation status.

We acknowledge that this study has some limitations to consider. We used self-reported PA and SB, which tends to be inaccurate as people usually overestimate their PA and underestimate their SB levels [101] compared to device measures, especially when using single item self-report to assess SB [102]. Nevertheless, self-report captures different constructs than device measure and currently remains the basis for physical activity guidelines [103]. Next to this, we had to deal with a high number of missing outcome data in Wave 2, likely caused by survey fatigue, as the participants had to fill out several questionnaires during their visit and the IPAQ was one of the last questionnaires to fill out. Multiple imputation made it possible to retain 628 out of the eligible 633 participants, and is becoming a standard practice to deal with missing data in cohort studies [104]. Furthermore, PA and SB are summed into an overall measure, disregarding the possible varying exposure effects on the separate domains of the outcome measures (e.g. leisure-time, commuting, occupational PA). Distinctive effects of greenness type and PA intensity (light, moderate and vigorous) and especially PA and SB modalities could be expected. These distributions of PA and SB by domain are expected to depend on educational status [105], with highly educated persons being more likely to occupy desk-based jobs [106] and hence display higher levels of occupational SB [107, 108]. Persons in manual labor tend to show higher levels of leisure time SB [109]. Future studies working on the association between (built) environment characteristics, PA and/or SB data should consider these modalities, as it is likely that they will show underlying relationships [110].

In this study, we used GIS software to collect objective environmental data, which is becoming the standard method for measuring environmental attributes. With the use of such spatial data, potential issues such as spatial dependency and heterogeneity could arise and need to be addressed. We assessed the relationship between greenness exposure, PA and SB by using a REWB model, which is a linear regression model that allows for multiple levels and is very suitable for analyzing longitudinal relationships between exposure and outcome [74]. However, such linear models assume that the association between exposure and outcome is homogeneous within spatial units and heterogeneous between spatial units, which can be troublesome. To overcome these issues, Feuillet et al. recently proposed to combine multilevel models with geographically weighted regression models [111]. This is potentially an important step forward in environmental health research.

Despite these limitations, this is one of the first studies to examine longitudinal associations between changes in greenness levels over time, including changes in greenness type and -diversity, with changing PA and SB levels. The results of this study create opportunities for replication studies, using objectively measured PA and SB data with sensors such as accelerometers.

## Conclusion

This study contributes to the growing, but still limited, longitudinal evidence of the effects of greenness exposure on PA and specifically SB. We found that, over a period of 9 years, an increase in TCD and in GLUM were associated with decreasing PA levels. No significant associations between greenness exposure and SB were observed. The need for greenness measures that capture different aspects of the green environment when investigating associations between greenness exposure and PA and SB levels is emphasized, as different aspects of green likely follow distinct pathways between exposure and behavior. Even though this study provided some contradicting results to the existing literature on greenness exposure and PA and SB, it provides novel evidence for the opposite effects of TCD on PA and SB among sexes, and varying strengths of the negative association between overall greenness and SB by NSES in the immediate green environment.

### Supplementary Information

Supplementary Material 1: **Image S1**. A visual represenation of the different greenness measures for Luxembourg City at study wave 1. **Image S2**. A visual represenation of street network buffers and built environment characteristics at study wave 1. **Table S1. **Correlation matrix of the three outcome measures and environmental covariates for the main buffer (1000m).** Table S2.** Summary of BDLTC building classification for both data collection periods. **Data imputation method specification**

Supplementary Material 2: **Table S1.** Description of the ORISCAV-LUX population and the current study population. **Table S2. **Description of participants’ individual and environmental characteristics by sex (n=628). **Table S3.** Description of participants’ individual and environmental characteristics by neighborhood SES (n=628). **Table S4.** Description of participants’ individual and environmental characteristics by lifestyle preference (n=628). **Table S5.** Description of participants’ individual and environmental characteristics by relocation status (n=628). **Table S6.** Greenness exposure and building density in all buffer sizes (500, 800, 1000m) (n=628).

Supplementary Material 3: **Table S1.** Results sensitivity analyses for greenness exposure and PA including interactions with sex (500m, 800m buffers).** Table S2.** Results sensitivity analyses for greenness exposure and SB including interactions with sex (500m, 800m buffers).** Table S3.** Results sensitivity analyses for greenness exposure and PA including interactions with NSES (500m, 800m buffers).** Table S4.** Results sensitivity analyses for greenness exposure and SB including interactions with NSES (500m, 800m buffers).** Table S5.** Results sensitivity analyses for greenness exposure and PA including interactions with lifestyle preference [for being active] (500m, 800m buffers).** Table S6.** Results sensitivity analyses for greenness exposure and SB including interactions with lifestyle preference [for being active] (500m, 800m buffers).** Table S7.** Results sensitivity analyses for greenness exposure and PA including interactions with relocation status (1000m buffer).** Table S8.** Results sensitivity analyses for greenness exposure and SB including interactions with relocation status (1000m buffer).** Table S9.** Results sensitivity analyses for greenness exposure and PA including interactions with relocation status (500m, 800m buffer).** Table S10.** Results sensitivity analyses for greenness exposure and SB including interactions with relocation status (500m, 800m buffer).** Figure S1.** Predicted PA and SB values by sex in the 800m buffer. **Figure S2.** Predicted SB values by neighborhood socio-economic status in the 500m buffer. **Figure S3. **Predicted PA and SB values by lifestyle preference [for being active] in the 500m buffer. **Figure S4. **Predicted SB values by lifestyle preference [for being active] in the 800m buffer. **Figure S5.** Predicted PA and SB values by relocation status in the 500m buffer.** Figure S6.** Predicted SB values by relocation status in the 800m buffer.

## Data Availability

De-identified data may be available upon reasonable request if consent is provided by all authors and the ORISCAV-Lux study group. Requests to access to the ORISCAV-LUX data should be directed to LM and requests to access to the MET’HOOD data to CP.

## Abbreviations

GIS: Geographic Information Systems
NCD: Non-communicable diseases
PA: Physical activity
SB: Sedentary behavior
NDVI: Normalized difference vegetation index
SES: Socioeconomic status
FE model: Fixed effect model
RE model: Random effect model
REWB model: Random-effect-within-between model
NSES: Neighborhood socioeconomic status
ORISCAV-LUX: Observation of Cardiovascular Risk Factors in Luxembourg Study
TCD: Tree cover density
SAVI: Soil-adjusted vegetation index
GLUM: Green land use mix
IPAQ: International physical activity questionnaire
MET-min: Metabolic equivalent per minute
MICE: Multiple imputation by chained equation
IQR: Interquartile range
UGS: Urban green space
