# Supplementary material for: Longitudinal study of changes in greenness exposure, physical activity and sedentary behavior in the ORISCAV-LUX cohort study
Source: Int J Health Geogr. 2024 May 21;23:14. doi: 10.1186/s12942-024-00374-7 (PMC11110334; doi:10.1186/s12942-024-00374-7)
Supplement: Supplementary file 1 — Supplementary Material 1: Image S1. A visual represenation of the different greenness measures for Luxembourg City at study wave 1. Image S2. A visual represenation of street network buffers and built environment characteristics at study wave 1. Table S1. Correlation matrix of the three outcome measures and environmental covariates for the main buffer (1000m). Table S2. Summary of BDLTC building classification for both data collection periods. Data imputation method specification [file 12942_2024_374_MOESM1_ESM.docx]

**Additional file 1**

**Image S1.** A visual represenation of the different greenness measures for Luxembourg City at study wave 1.

**Luxembourg City**


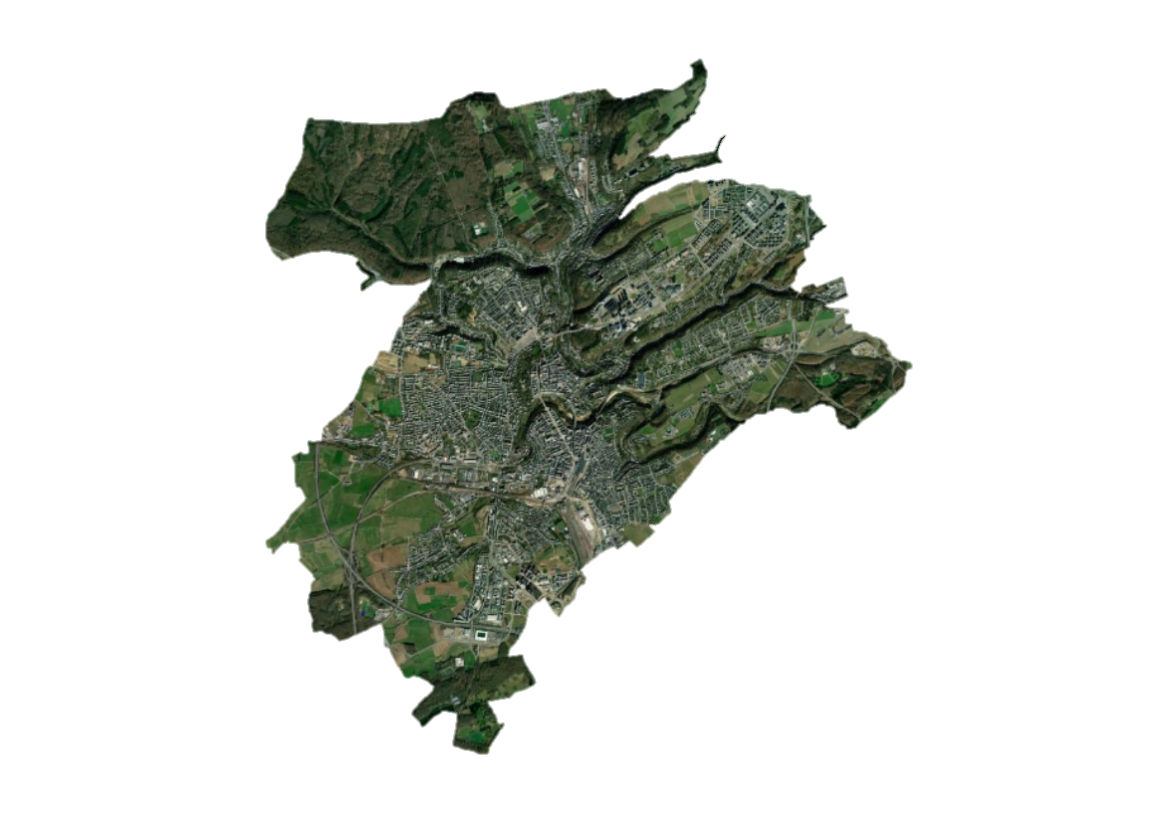

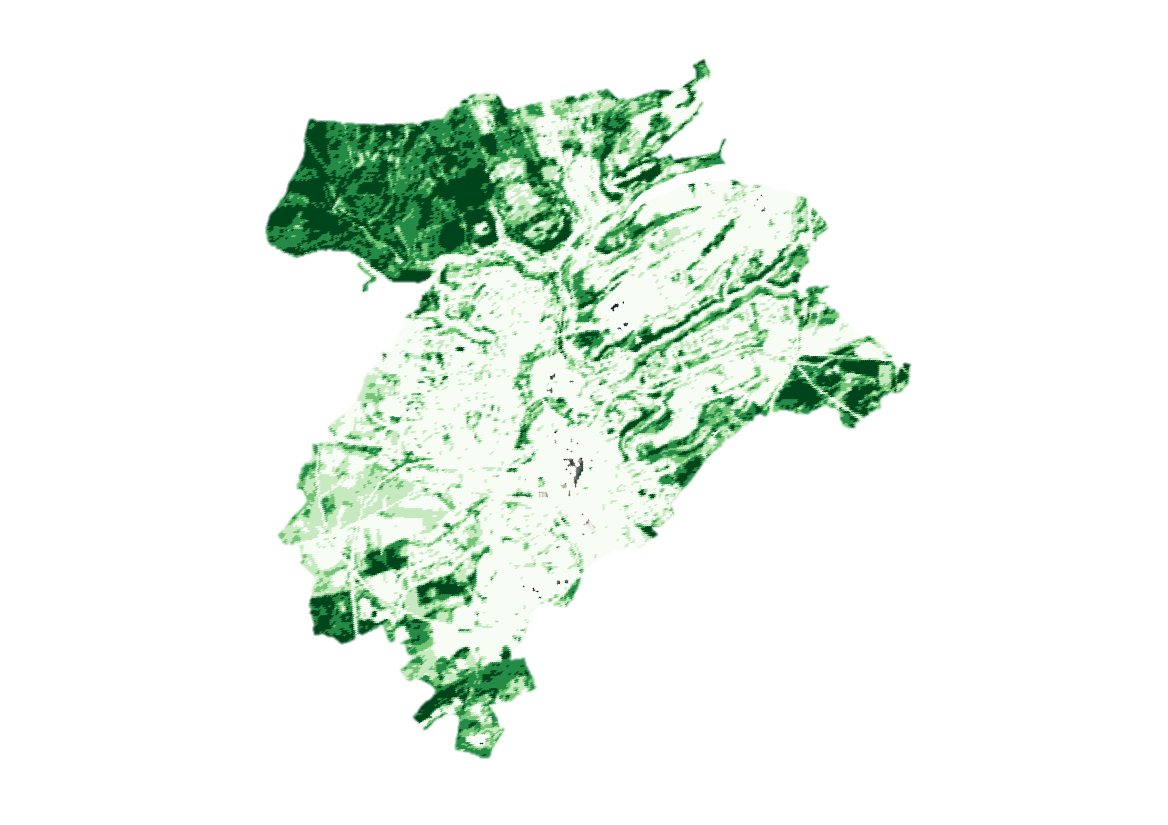


a) Hybrid photo b) Soil-adjusted vegetation index (SAVI)


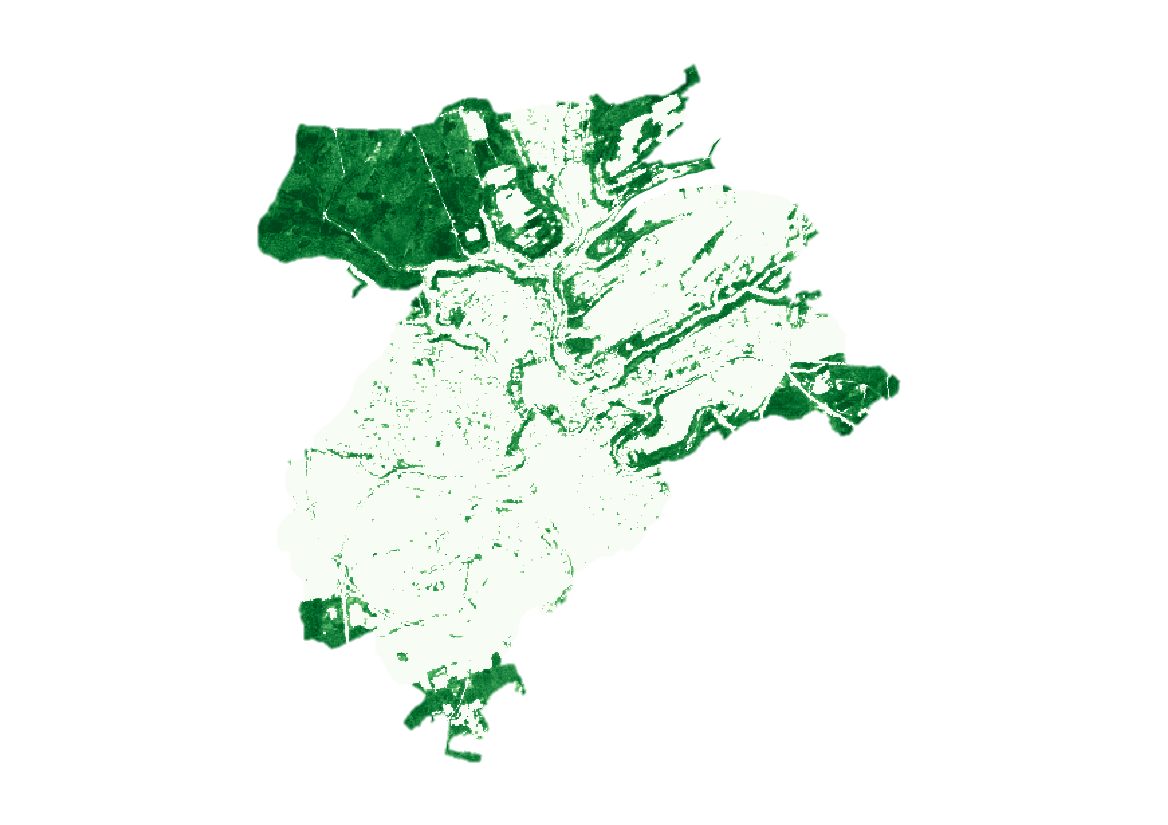

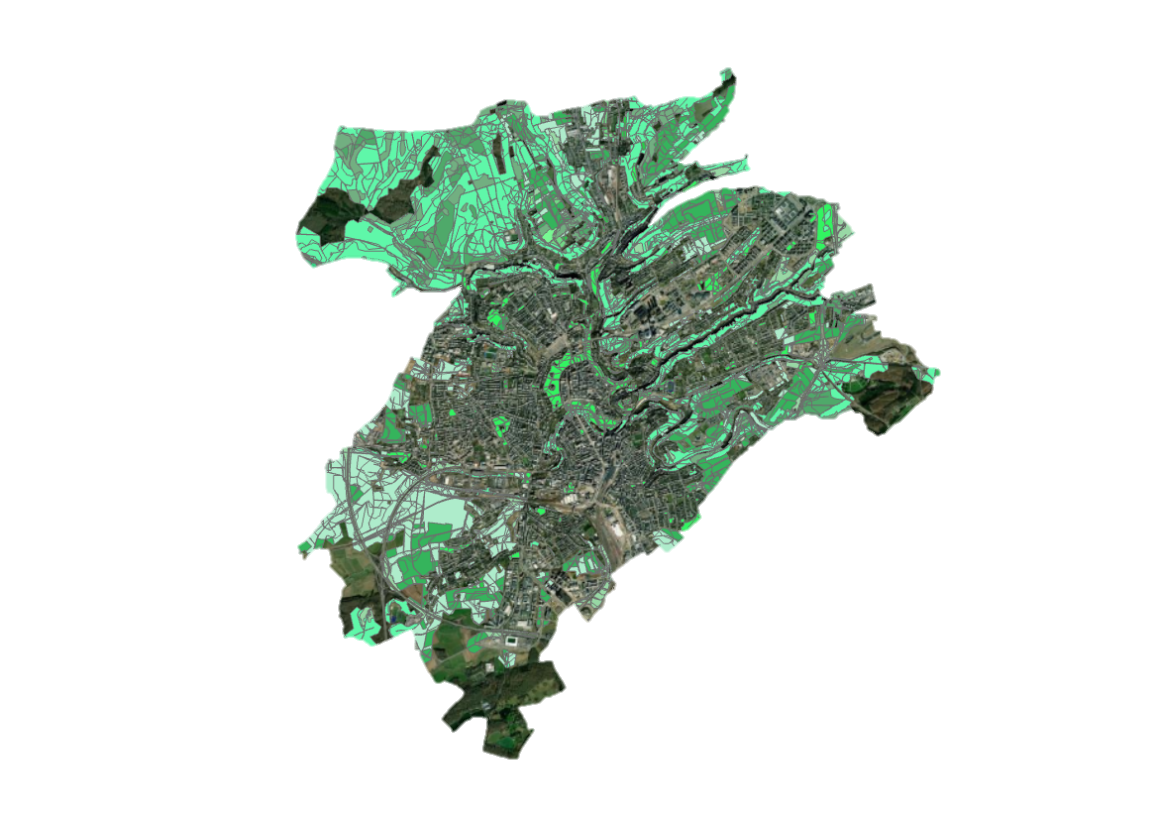


c) Tree cover density (TCD) d) Green land use mix (GLUM)


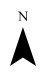

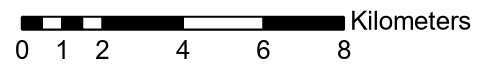


**Image S2.** A visual represenation of street network buffers and built environment characteristics at study wave 1.


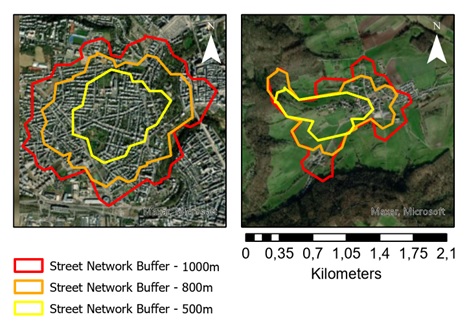


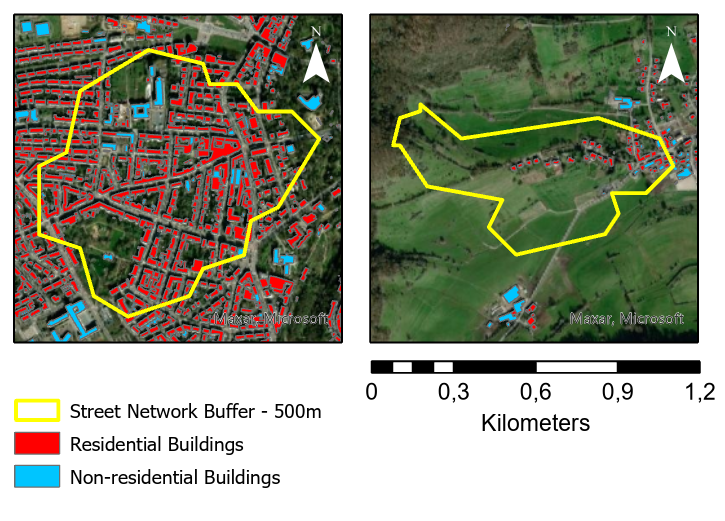


**Above:** the three different street network buffer sizes for participants living in an urban (left) and rural (right) area, **below:** built environment characteristics for participants living in an urban (left) and rural (right) area.

**Table S1**. Spearman correlation matrix of the three outcome measures and environmental covariates for the main buffer (1000m).

|  | TCD | SAVI | GLUM | Residential Building Density | Nonresidential Building Density | Housing Price |
| --- | --- | --- | --- | --- | --- | --- |
| TCD | 1,0000 |  |  |  |  |  |
| SAVI | 0,3362 | 1,0000 |  |  |  |  |
| GLUM | 0,3982 | 0,7095 | 1,0000 |  |  |  |
| Residential Building Density | -0,0528 | -0,7318 | -0,7311 | 1,0000 |  |  |
| Nonresidential Building Density | -0,2287 | -0,7279 | -0,6658 | 0,6145 | 1,0000 |  |
| Housing Price | -0,1756 | -0,3889 | -0,1973 | 0,2453 | 0,2042 | 1,0000 |

TCD = tree cover density, SAVI = soil adjusted vegetation index, and GLUM = green land use mix

**Table S2.** Summary of BDLTC building classification for both data collection periods

|  | **BDLTC 2008** | |  | **BDLTC 2018** | | |
| --- | --- | --- | --- | --- | --- | --- |
| 1 | **4.5 BATIMENT** | bâtiment autre que ceux distingués par la suite |  | **4.1 BATIMENT** | | |
| 2 | **4.6 BATI_INDUS** | bâtiment d'activité industrielle ou artisanale |  | **attribut** | **Nature** | **commentaire** |
| 3 | **4.7 BATI_AGRI** | bâtiment d'activité agricole | 1 | NULL | Bâtiment ordinaire | Bâtiment ordinaire, habitation, et construction à l’architecture ordinaire (toit en tuile ou en ardoise) ainsi que les bâtiments en construction. |
| 4 | **4.8 BATI_COMME** | bâtiment d'activité commerciale | 2 | 10000 | Bâtiment industriel | Bâtiment d’activité industrielle ou artisanale. Regroupe les hangars, ateliers, usines. |
|  | **4.9 BATI_PUB** | bâtiment à caractère public ou administratif | 3 | 10001 | Centre de recyclage | Centre de recyclage |
| 5 | 0 | Non codé | 4 | 10002 | Station d’épuration | Station d’épuration |
| 6 | 1 | Hotel de ville | 5 | 20000 | Bâtiment agricole | bâtiment ayant une fonction de production agricole : élevage, laiterie, silo. Les fermes sont à traiter en bâtiment ordinaire. |
| 7 | 2 | Mairie | 6 | 30000 | Bâtiment commercial | bâtiments commerciaux à l’architecture caractéristique (toit en tôle ou fibrociment). Un commerce de surface inférieure à 500 m² donc l’architecture est ordinaire est traité en bâtiment ordinaire. |
| 8 | 3 | Gendarmerie | 7 | 40000 | Bâtiment publique | Bâtiment publique ne répondant pas aux cas listés cidessous. |
| 9 | 4 | Police | 8 | 40101 | Gendarmerie | Gendarmerie |
| 10 | 5 | Ministère | 9 | 40102 | Police | Bâtiment de gendarmerie accueillant le public |
| 11 | 6 | Administration | 10 | 40103 | Douane | Centre douanier. |
| 12 | 7 | Etablissement public | 11 | 40201 | Palais de justice | Palais de justice |
| 13 | 8 | Centre de télécommunications | 12 | 40202 | Centre pénitentiaire | Centre pénitentiaire |
| 14 | 9 | Bureau de Poste | 13 | 40301 | Hôtel de ville | Hôtel de ville |
| 15 | 10 | Centre d'intervention | 14 | 40302 | Mairie | Mairie |
| 16 | 11 | Centre douanier | 15 | 40303 | Ministère | Ministère |
| 17 | 12 | Institutions européennes | 16 | 40304 | Institution européenne | Institution européenne |
| 18 | 13 | Palais Grand-Ducal | 17 | 40305 | Autre administration | Autre administration accueillant du public. |
| 19 | 14 | Palais de justice | 18 | 40401 | Bureau de poste | Bureau de poste |
| 20 | 15 | Etablissement pénitentiaire | 19 | 40402 | Établissement publique | Établissement publique accueillant du public. |
| 21 | 16 | Enceinte militaire | 20 | 40501 | Bâtiment militaire | Bâtiment situé dans une enceinte militaire. |
| 22 | 17 | Stand de tir militaire | 21 | 40502 | Stand de tir militaire | Stand de tir militaire |
| 23 | 18 | Aérogare | 22 | 40503 | Centre d’instruction militaire | Centre d’instruction militaire |
| 24 | 19 | Ecole | 23 | 40601 | Centre d’intervention | Bâtiment principal de centre de secours, caserne de pompiers. |
|  | 20 |  | 24 | 40701 | École, collège, lycée, université | École, collège, lycée, université |
|  | 21 |  | 25 | 40702 | Maison d’enfants | Maison d’enfants |
| 25 | 22 | Maison d'enfants | 26 | 40703 | Centre d’éducation différenciée | Centre d’éducation différenciée |
| 26 | 23 | Centre d'education differenciee | 27 | 40704 | Conservatoire de musique | Conservatoire de musique |
| 27 | 24 | Conservatoire de musique | 28 | 40705 | Garderie | Garderie |
| 28 | 25 | Centre culturel | 29 | 40706 | Centre socio-éducatif | Centre socio-éducatif |
| 29 | 26 | Théatre | 30 | 40801 | Gare | Gare ferroviaire, bâtiment accueillant le publique  (renseignement, vente de billet, …) |
| 30 | 27 | Musée | 31 | 40802 | Aérogare | Bâtiment accueillant le public, vente, embarquement, débarquement, … |
| 31 | 28 | Bibliothèque | 32 | 40803 | Centre de contrôle technique automobile | Centre de contrôle technique automobile |
| 32 | 29 | Hôpital | 33 | 40901 | Bâtiment de camping | Accueil, sanitaires, … |
| 33 | 30 | Maison de soins | 34 | 40902 | Auberge de jeunesse | Auberge de jeunesse |
| 34 | 31 | Maison de retraite | 35 | 41001 | Théâtre | Théâtre |
| 35 | 32 | Maison des aveugles | 36 | 41002 | Musée | Musée |
| 36 | 33 | Sanatorium | 37 | 41003 | bibliothèque | bibliothèque |
| 37 | 34 | Foyer | 38 | 41004 | Site historique | Site historique |
| 38 | 35 | Croix-Rouge | 39 | 41005 | Casemate | Casemate |
| 39 | 36 | Etablissement thermal | 40 | 41006 | Centre d’activités culturelles | Centre d’activités culturelles |
| 40 | 37 | Auberge de jeunesse | 41 | 41101 | Bâtiment de parc public | Bâtiment de parc public accueillant du public |
| 41 | 38 | Parc public | 42 | 41102 | Foire | Bâtiments accueillant du public dans une foire ou un centre d’exposition |
| 42 | 39 | Camping | 43 | 41103 | Centre de loisir | Bâtiments accueillant du public inclus dans un centre de loisir |
| 43 | 40 | Contrôle technique des automobiles | 44 | 41201 | Hôpital | Hôpital |
| 44 | 41 | Foire | 45 | 41202 | Maison de soin | Maison de soin |
| 45 | 42 | Centre de loisirs | 46 | 41203 | Sanatorium | Sanatorium |
| 46 | 43 | Site historique | 47 | 41204 | Établissement thermal | Établissement thermal |
| 47 | 44 | Casemate | 48 | 41205 | Croix rouge | Croix rouge |
| 48 | 45 | Garderie | 49 | 41206 | Foyer | Foyer |
|  | 46 |  | 50 | 41207 | Maison de retraite | Maison de retraite |
|  | 47 |  | 51 | 41208 | Maison des aveugles | Maison des aveugles |
|  | 48 |  | 52 | 41301 | Usage mixte | Bâtiment accueillant du public répondant à plusieurs des critères énumérés. |
|  | 49 |  | 53 | 41302 | Kiosque | Kiosque |
| 49 | 50 | Usage mixte | 54 | 41303 | Lavoir | Lavoir |
| 50 | **4.10 SERRE** | bâtiment vitré destiné à l'horticulture | 55 | 41304 | Centre de télécommunication | Centre de télécommunication |
| 51 | **4.11 CONS_LEGER** | bâtiment vitré destiné à l'horticulture | 56 | 41305 | Palais grand-ducal | Palais grand-ducal |
| 52 | **4.13 BATI_REMAR** | bâtiment remarquable dans le paysage | 57 | 50001 | Cathédrale | Cathédrale |
|  | **4.17 BATI_RELIG** | | 58 | 50002 | Église | Église |
| 53 | 0 | Non codé | 59 | 50003 | Temple protestant | Temple protestant |
| 54 | 1 | Cathédrale | 60 | 50004 | Église orthodoxe russe | Église orthodoxe russe |
| 55 | 2 | Eglise | 61 | 50005 | Chapelle | Chapelle |
| 56 | 3 | Eglise protestante | 62 | 50006 | Oratoire | Oratoire |
| 57 | 4 | Eglise orthodoxe russe | 63 | 50007 | Couvent | Couvent |
| 58 | 5 | Chapelle | 64 | 50008 | Abbaye | Abbaye |
| 59 | 6 | Oratoire | 65 | 50009 | Grotte à caractère religieux | Grotte à caractère religieux |
| 60 | 7 | Couvent | 66 | 50010 | Séminaire | Séminaire |
| 61 | 8 | Abbaye | 67 | 50011 | Synagogue | Synagogue |
| 62 | 9 | Grotte | 68 | 50012 | Morgue | Morgue |
| 63 | 10 | Séminaire | 69 | 60000 | Bâtiment remarquable | bâtiment ayant une architecture qui les distingue (moulin, tour, donjon, château, …) |
| 64 | 11 | Synagogue | 70 | 70001 | Piscine | Piscine |
| 65 | 12 | Morgue | 71 | 70002 | Centre omnisport | Centre omnisport |
|  | **4.22 CENT_SPORT** | | 72 | 80000 | Serre | Serre de construction pérenne avec armature maçonnée et vitres |
| 66 | 0 | Omnisport | 73 | 90000 | Construction légère | Cabane, baraque, abris permanent |
| 67 | 1 | Piscine | 74 | 100000 | Bâtiment parking | Immeuble de parking réservé uniquement à cet usage. |
| 68 | **2.2 GARE** | | 75 | 110000 | Bâtiment tertiaire | Bâtiment à usage tertiaire signalé par l’ACT. |
|  |  | | 76 | 50000 | Batiment religieus | Non codé |

**Data imputation method specification**

Two full ORISCAV datasets were used for data imputations (1432 respondents in Wave 1, 1558 respondents in Wave 2). At the first stage, data cleaning and variable reformatting were performed. Namely, the following cleaning steps were conducted:

1. Vegetable and fruit consumption was summarised as variables *tot_vegfr_1* (Wave 1) and *tot_vegfr_2* (Wave 2)
2. Variable *ALCOOL_J_V2* was categorised into *ALCOOL_S_V2* (Wave 2)
3. IPAQ time variables were summarised as *APINTHE_T, APMODHE_T, APMARHE_T, APASSH_T* (Wave 1) and *APINTHE_T_V2, APMODHE_T_V2, APMARHE_T_V2, APASSH_T_V2* (Wave 2)
4. Faulty IPAQ data was either set to NA or truncated, in accordance with the “IPAQ – Short form” data cleaning guide
5. Sedentary behaviour time variables were combined into *TVSEMH_T, TVREPH_T, ORDSEMH_T, ORDREPH_T* (Wave 1) and *TVSEMH_T_V2, TVREPH_T_V2, ORDSEMH_T_V2, ORDREPH_T_V2* (Wave 2)
6. Faulty Sedentary Behaviour data, exceeding the total limit of 16h per day was set to NAs
7. *ecran_travail, ecran_repos* were excluded from the dataset, as they were equal to *TVSEMH_T+ORDSEMH_T* and *TVREPH_T+ORDREPH_T* correspondingly
8. *ECRAN_TRAVAIL_V2, ECRAN_REPOS_V2* were excluded from the dataset, as they were equal to *TVSEMH_T_V2* and *TVREPH_T_V2* correspondingly

The proportions of complete cases of IPAQ Wave 1, Sedentary Behaviour Wave 1, IPAQ Wave 2, and Sedentary Behaviour Wave 2 were 94%, 97%, 56%, and 44% correspondingly. Therefore, the largest proportion of missing data among all the parts was 56%. Thereof, the number of copies of the imputed datasets was set to 60 (>56).

The following list of variables was tested for correlation/association with IPAQ variables, Sedentary Behaviour variables and *APASSH_T* variable in Wave 1: *SM_obesity_ATP3, Tryg_ATP3, HDL_ATP3, BP_ATP3, diabete_ATP3, SM_ATP3, Plasma, gluc, hemgl, insu, choltot, hdl, ldl, trig, crea, crp, apoa, apob, aciur, got, gpt, gamm, PAS, PAD, bmi, NRJ, tot_vegfr_1, Q59, pratsport, sexe, age, European_country, marital_status, dip_c3, Activite_cat, work_status, revenu_equivalent_adulte, nbe, distribfumeurs,alcohol_consumption, sante_c, antper1, antper2,antper3, antper4, antper5, antper6, antper7, antper8, antper9, antper10, antper11, antper12,antper13, antper14, dorm, alim, poids, soci, stress, actiphy, fumee, HOMA_IR, creauri, leuco, eryth, plaq, hemo, hema, pdsv, taiv, ttaiv, thanv, Ressource_perception, score_morbidite,*

The following list of variables was tested for correlation/association with IPAQ variables, Sedentary Behaviour variables and *APASSH_T_V2* variable in Wave 2: *SM_OBESITY_ATP3_V2, TRYG_ATP3_V2, HDL_ATP3_V2, BP_ATP3_V2, DIABETE_ATP3_V2, SM_ATP3_V2, HOMA_IR_V2, PLASMA_V2, GLUC_N_V2, HEMGL_N_V2, INSU_N_V2, CHOLTOT_N_V2, HDL_N_V2, LDL_N_V2, TRIG_N_V2, CREA_N_V2, CREAURI_N_V2, CRP_N_V2, APOA_N_V2, APOB_N_V2, ACIUR_N_V2, GOT_N_V2, GPT_N_V2, GAMM_N_V2, PAS_V2, PAD_V2, WBC_N_V2, RBC_N_V2, PLAQ_N_V2, HGB_N_V2, HCT_N_V2, IMC_V2, POIDS_V2, TAIV_V2, TTAIV_V2, THANV_V2, NRJ_V2, tot_vegfr_2, Q59_V2, PRATSPOR_V2, SEXE_V2, AGE_V2, EUROPEAN_COUNTRY_V2, MARITAL_STATUS_V2, DIP_C3_V2, ACTIVITE_CAT_V2, WORK_STATUS_V2, RESSOURCE_PERCEPTION_V2, REVENU_EQUIVALENT_ADULTE_V2, NBE_V2, DISTRIBFUMEURS_V2, ALCOOL_J_V2, SANTE_PERCU_V2, SCORE_MORBIDITE_V2, INS_CARDIAQUE_V2, TROUBLE_RYTHME_V2, ANGINE_POIT_V2, INFARCTUS_V2, VALVES_V2, AVC_V2, HYPERTENSION_V2, ARTERITE_V2, VARICES_V2, ULCERE_V2, DOULEUR_POIT_V2, DIABETE_1_V2, DIABETE_2_V2, DYSLIPID_V2, DORM_V2, ALIM_V2, POIDS_QOL_V2, SOCIALE_V2, STRESS_V2, ACTIPHY_V2, FUMEE_V2, RATIO_TAILLE_HANCHE_V2*, AD_ig_gradient_ENMO_0.24hr, AD_mean_ENMO_mg_0.24hr, AD_M2_ENMO_mg_0.24hr, AD_M0.5_ENMO_mg_0.24hr, AD_M0.25_ENMO_mg_0.24hr, dur_day_MVPA_bts_10_min_pla, dur_day_IN_bts_30_min_pla, dur_day_total_IN_min_pla, dur_day_total_LIG_min_pla, dur_day_total_MOD_min_pla, dur_day_total_VIG_min_pla, dur_day_total_MVPA_min_pla, power_law_exponent_alpha_SED, median_bout_length_s_SED, gini_index_SED.

A continuous variable was included in an imputation model if its Pearson correlation coefficient with the imputed section of the questionnaire was > 0.15 for Wave 2 (>0.4 for Wave 1), its Biserial correlation coefficient was >0.3 (for dichotomous variables) or its square root of Eta-square was >0.2 (for nominal variables). If two auxiliary variables that have demonstrated multi-collinearity, the one in the pair of such variables that had lower correlation/association with the imputed variable, was not included in an imputation model.

The following variables satisfied one of the conditions above and were used as auxiliary variables for imputations:

| Imputed section | Auxiliary variables |
| --- | --- |
| IPAQ 1 | - |
| *APASSH_T* | *dip_c3, Activite_cat, European_country* |
| Sedentary Behaviour | *Activite_cat* |
| IPAQ 2 | *EUROPEAN_COUNTRY_V2, ACTIVITE_CAT_V2, AGE_V2, PAS_V2, dur_day_MVPA_bts_10_min_pla, dur_day_total_MVPA_min_pla, AD_mean_ENMO_mg_0.24hr* |
| *APASSH_T_V2* | *IP_C3_V2, ACTIVITE_CAT_V2, AGE_V2, TAIV_V2, REVENU_EQUIVALENT_ADULTE_V2, dur_day_total_LIG_min_pla, dur_day_IN_bts_30_min_pla, dur_day_total_IN_min_pla* |
| Sedentary Behaviour 2 | *CREAURI_N_V2, dur_day_total_LIG_min_pla, AD_mean_ENMO_mg_0.24hr, dur_day_IN_bts_30_min_pla, dur_day_total_IN_min_pla* |

All other potential auxiliary variables had very low association/correlation with the imputed sections, and were not used in imputation models, as their inclusion would not have benefitted in terms of estimates accuracy or bias reduction.

After applying MICE (alternatively named Fully Conditional Specification), there were 60 imputed datasets. The method used for every variable was PMM (Predictive Mean Matching).

Output files:

*the variable *.imp* represents to which data set each row belongs (0 – original dataset, 1-60 – imputed datasets)
**all newly created and imputed variables are located at the end of the data frames

Wave 1: *imp_w1_tot_apr* – 60 imputed datasets, plus the initial dataset in the beginning (top) of the data frame (in SPSS format ready for analyses) – 1432 observations per dataset
**If first 1432 rows are deleted, then the data frame is in same format as in SAS, prepared for analyses and pooling.**

Wave 2: *imp_w2_tot_apr* – 60 imputed datasets, with the initial dataset in the beginning (top) of the data frame (in SPSS format ready for analyses) – 1438 observations per dataset

**If first 1558 rows are deleted, then the data frame is in same format as in SAS.**

Wave1 + Wave 2: *imp_tot_IPAQ_apr* – 60 imputed datasets, with the initial dataset in the beginning (top) of the data frame (in SPSS format ready for analyses) – 633 observations per dataset, only MET’HOOD patients

**If first 633 rows are deleted, then the data frame is in same format as in SAS.**
