# Supplementary material for: Longitudinal study of changes in greenness exposure, physical activity and sedentary behavior in the ORISCAV-LUX cohort study
Source: Int J Health Geogr. 2024 May 21;23:14. doi: 10.1186/s12942-024-00374-7 (PMC11110334; doi:10.1186/s12942-024-00374-7)
Supplement: Supplementary file 2 — Supplementary Material 2: Table S1. Description of the ORISCAV-LUX population and the current study population. Table S2. Description of participants’ individual and environmental characteristics by sex (n=628). Table S3. Description of participants’ individual and environmental characteristics by neighborhood SES (n=628). Table S4. Description of participants’ individual and environmental characteristics by lifestyle preference (n=628). Table S5. Description of participants’ individual and environmental characteristics by relocation status (n=628). Table S6. Greenness exposure and building density in all buffer sizes (500, 800, 1000m) (n=628). [file 12942_2024_374_MOESM2_ESM.docx]

**Additional file 2**

**Description of sociodemographic characteristics the ORISCAV-LUX I study population and the current study sample.**

| **Table S1.** Sociodemographics of the ORISCAV-LUX I population and the final study sample. | | | | | |
| --- | --- | --- | --- | --- | --- |
|  |  | **ORISCAV-LUX study I**  **(n = 1432)** | **missing** | **Final sample**  **(n = 628)** | **missing** |
| **Individual-level characteristics** |  |  |  |  |  |
| Age (years), mean ± SD |  | 44.3 ± 13.1 |  | 44.05 ± 11.97 |  |
| Sex, n (%) |  |  |  |  |  |
| Women |  | 735 (51.3) |  | 309 (49.20) |  |
| Men |  | 697 (49.7) |  | 319 (50.80) |  |
| Marital status, n (%) |  |  |  |  |  |
| Married/living with partner |  | 997 (69.6) |  | 468 (74.41) |  |
| Single/never married |  | 271 (18.9) |  | 100 (16.11) |  |
| Divorced/separated/widowed |  | 164 (11.5) |  | 60 (9.48) |  |
| Education level, n (%) |  |  | 14 |  | 4 |
| No diploma |  | 380 (26.8) |  | 107 (17.01) |  |
| Secondary education |  | 667 (47.0) |  | 305 (49.28) |  |
| Tertiary education |  | 371 (26.2) |  | 212 (33.70) |  |
| Lifestyle preference - Importance of PA, n (%) |  |  | 2 |  |  |
| Important |  | 1127 (78.8) |  | 518 (82.48) |  |
| Little to no importance |  | 303 (21.2) |  | 110 (17.52) |  |

**Descriptive results for each group (sex, neighborhood SES, relocation)**

| **Table S2.** Description of participants’ individual and environmental characteristics by sex (n=628) | | | | | | | | | | | | | | | |
| --- | --- | --- | --- | --- | --- | --- | --- | --- | --- | --- | --- | --- | --- | --- | --- |
| Variables | Mean ± SD or Proportion (%) | | | | | | | | | | | | | | |
|  | MET’HOOD 1 | | | | | | |  | MET’HOOD 2 | | | | | | |
|  | n |  | *#*  *missing* |  | n |  | *#*  *missing* |  | n |  | *#*  *missing* |  | n |  | *#*  *missing* |
|  | *Men (n = 319)* | | |  | *Women (n = 309)* | | |  | *Men (n = 319)* | | |  | Women (n = 309) | | |
| **Individual-level characteristics** | | | | | | | | | | | | | | | |
| **Age** |  | **43.4 ± 12.2** |  |  |  | **44.8 ± 11.7** |  |  |  | **51.3 ± 12.1** |  |  |  | **52.7 ± 11.6** |  |
| **Marital status** | **319** |  |  |  | **309** |  |  |  | **319** |  |  |  | **309** |  |  |
| Married / Living with partner | 242 | 75.86 |  |  | 226 | 73.14 |  |  | 256 | 80.25 |  |  | 231 | 74.76 |  |
| Single | 57 | 17.87 |  |  | 43 | 13.92 |  |  | 34 | 10.66 |  |  | 28 | 9.06 |  |
| Divorced / Separated / Widowed | 20 | 6.27 |  |  | 40 | 12.94 |  |  | 29 | 9.09 |  |  | 50 | 16.18 |  |
| **Education** | **316** |  | **3** |  | **308** |  | **1** |  | **315** |  | **4** |  | **308** |  | **1** |
| No diploma | 53 | 16.77 |  |  | 54 | 17.53 |  |  | 41 | 13.02 |  |  | 40 | 12.99 |  |
| Secondary level | 155 | 49.05 |  |  | 150 | 48.70 |  |  | 145 | 46.03 |  |  | 145 | 47.08 |  |
| University level | 108 | 34.18 |  |  | 104 | 33.77 |  |  | 129 | 40.95 |  |  | 123 | 39.94 |  |
| **Lifestyle preference - Importance of PA** | **319** |  |  |  | **309** |  |  |  | **319** |  |  |  | **309** |  |  |
| Important | 263 | 82.45 |  |  | 255 | 82.52 |  |  | 269 | 84.33 |  |  | 267 | 86.41 |  |
| Little to no importance | 56 | 17.55 |  |  | 54 | 17.48 |  |  | 50 | 15.67 |  |  | 42 | 13.59 |  |
| **Season (questionnaire completion date)** | **319** |  |  |  | **309** |  |  |  | **314** |  |  |  | **309** |  |  |
| Spring (March, April, May) | 91 | 28.53 |  |  | 80 | 25.89 |  |  | 86 | 27.39 |  |  | 86 | 27.83 |  |
| Summer (June, July, August) | 50 | 15.67 |  |  | 53 | 17.15 |  |  | 91 | 28.98 |  |  | 91 | 29.45 |  |
| Autumn (September, October, November) | 80 | 25.08 |  |  | 70 | 22.65 |  |  | 58 | 18.47 |  |  | 53 | 17.15 |  |
| Winter (December, January, February) | 98 | 30.72 |  |  | 106 | 34.30 |  |  | 79 | 25.16 |  |  | 79 | 25.57 |  |
| **Environmental-level characteristics** | | | | | | | | | | | | | | | |
| **Relocation status** |  |  |  |  |  |  |  |  | **319** |  |  |  | **309** |  |  |
| Non mover |  |  |  |  |  |  |  |  | 209 | 65.52 |  |  | 224 | 72.49 |  |
| Relocated |  |  |  |  |  |  |  |  | 110 | 34.48 |  |  | 85 | 27.51 |  |
| **Average housing price (sq m)** |  | **3436**  **(3190-4087)** |  |  |  | **3497**  **(3221-4107)** |  |  |  | **4608**  **(4265-5715)** |  |  |  | **4651**  **(4298-5715)** |  |
| **Building density (0-1)** |  |  |  |  |  |  |  |  |  |  |  |  |  |  |  |
| Residential buildings |  | 0.075 ± 0.053 |  |  |  | 0.070 ± 0.047 |  |  |  | 0.075 ± 0.050 |  |  |  | 0.071 ± 0.045 |  |
| Non-residential buildings |  | 0.025 ± 0.020 |  |  |  | 0.025 ± 0.018 |  |  |  | 0.029 ± 0.022 |  |  |  | 0.028 ± 0.023 |  |
| **Greenness (0-1)** |  |  |  |  |  |  |  |  |  |  |  |  |  |  |  |
| Tree cover density |  | 0.17 ± 0.10 |  |  |  | 0.16 ± 0.11 |  |  |  | 0.14 ± 0.10 |  |  |  | 0.13 ± 0.11 |  |
| Soil-adjusted vegetation index |  | 0.42 ± 0.09 |  |  |  | 0.42 ± 0.08 |  |  |  | 0.37 ± 0.06 |  |  |  | 0.37 ± 0.06 |  |
| Green land use mix |  | 0.31 ± 0.11 |  |  |  | 0.31 ± 0.10 |  |  |  | 0.31 ± 0.10 |  |  |  | 0.313 ± 0.10 |  |
| **Outcomes** | | | | | | | | | | | | | | | |
| MET-minutes per week |  | 3546  (1508-6078) |  |  |  | 3850  (2076-6510) |  |  |  | 3512  (1511-6974) |  |  |  | 3492  (1282-6873) |  |
| Sitting time per day |  | 420 (270-600) |  |  |  | 360 (240-480) |  |  |  | 360 (240-525) |  |  |  | 300  (180-480) |  |

| **Table S3.** Description of participants’ individual and environmental characteristics by neighborhood SES (n=628) | | | | | | | | | | | | | | | |
| --- | --- | --- | --- | --- | --- | --- | --- | --- | --- | --- | --- | --- | --- | --- | --- |
| Variables | Mean ± SD or Proportion (%) | | | | | | | | | | | | | | |
|  | MET’HOOD 1 | | | | | | |  | MET’HOOD 2 | | | | | | |
|  | n |  | *#*  *missing* |  | n |  | *#*  *missing* |  | n |  | *#*  *missing* |  | n |  | *#*  *missing* |
|  |  |  |  |  |  |  |  |  |  |  |  |  |  |  |  |
|  | *No to little change (n = 319)* | | |  | *Increase (n = 309)* | | |  | *No to little change (n = 319)* | | |  | *Increase (n = 309)* | | |
| **Individual-level characteristics** | | | | | | | | | | | | | | | |
| **Age** |  | **43.8 ± 12.5** |  |  |  | **44.3 ± 11.4** |  |  |  | **51.8 ± 12.4** |  |  |  | **52.2 ± 11.3** |  |
| **Sex** | **319** |  |  |  | **309** |  |  |  | **319** |  |  |  | **309** |  |  |
| Men | 173 | 54.23 |  |  | 146 | 47.25 |  |  | 173 | 54.23 |  |  | 146 | 47.25 |  |
| Women | 146 | 45.77 |  |  | 163 | 52.75 |  |  | 146 | 45.77 |  |  | 163 | 52.75 |  |
| **Marital status** | **319** |  |  |  | **309** |  |  |  | **319** |  |  |  | **309** |  |  |
| Married / Living with partner | 238 | 74.61 |  |  | 230 | 74.43 |  |  | 252 | 79.00 |  |  | 235 | 76.05 |  |
| Single | 52 | 16.30 |  |  | 48 | 15.53 |  |  | 31 | 9.72 |  |  | 31 | 10.03 |  |
| Divorced / Separated / Widowed | 29 | 9.09 |  |  | 31 | 10.03 |  |  | 36 | 11.29 |  |  | 43 | 13.92 |  |
| **Education** | **318** |  | **1** |  | **306** |  | **3** |  | **317** |  | **2** |  | **306** |  | **3** |
| No diploma | 69 | 21.70 |  |  | 38 | 12.42 |  |  | 55 | 17.35 |  |  | 26 | 8.50 |  |
| Secondary level | 165 | 51.89 |  |  | 140 | 45.75 |  |  | 155 | 48.90 |  |  | 135 | 44.12 |  |
| University level | 84 | 26.42 |  |  | 128 | 41.83 |  |  | 107 | 33.75 |  |  | 145 | 47.39 |  |
| **Lifestyle preference - Importance of PA** | **319** |  |  |  | **309** |  |  |  | **319** |  |  |  | **309** |  |  |
| Important | 258 | 80.88 |  |  | 260 | 84.14 |  |  | 266 | 83.39 |  |  | 270 | 87.38 |  |
| Little to no importance | 61 | 19.12 |  |  | 49 | 15.86 |  |  | 53 | 16.61 |  |  | 39 | 12.62 |  |
| **Season (questionnaire completion date)** | **319** |  |  |  | **309** |  |  |  | **319** |  |  |  | **309** |  |  |
| Spring (March, April, May) | 64 | 20.06 |  |  | 107 | 34.63 |  |  | 89 | 27.90 |  |  | 91 | 29.45 |  |
| Summer (June, July, August) | 58 | 18.18 |  |  | 45 | 14.56 |  |  | 88 | 27.59 |  |  | 96 | 31.07 |  |
| Autumn (September, October, November) | 98 | 30.72 |  |  | 52 | 16.83 |  |  | 67 | 21.00 |  |  | 44 | 14.24 |  |
| Winter (December, January, February) | 99 | 31.03 |  |  | 105 | 33.98 |  |  | 75 | 23.51 |  |  | 78 | 25.24 |  |
| **Environmental-level characteristics** | | | | | | | | | | | | | | | |
| **Relocation status** |  |  |  |  |  |  |  |  | **319** |  |  |  | **309** |  |  |
| Non mover |  |  |  |  |  |  |  |  | 222 | 69.59 |  |  | 211 | 68.28 |  |
| Relocated |  |  |  |  |  |  |  |  | 97 | 30.41 |  |  | 98 | 31.72 |  |
| **Average housing price (sq m)** |  | **3372 (3149-3531)** |  |  |  | **3888 (3405-4258)** |  |  |  | **4298 (4038-4508)** |  |  |  | **5715 (5042-7074)** |  |
| **Building density** |  |  |  |  |  |  |  |  |  |  |  |  |  |  |  |
| Residential buildings |  | 0.070 ± 0.052 |  |  |  | 0.075 ± 0.048 |  |  |  | 0.068 ± 0.050 |  |  |  | 0.078 ± 0.045 |  |
| Non-residential buildings |  | 0.023 ± 0.018 |  |  |  | 0.026 ± 0.019 |  |  |  | 0.026 ± 0.021 |  |  |  | 0.031 ± 0.024 |  |
| **Greenness** |  |  |  |  |  |  |  |  |  |  |  |  |  |  |  |
| Tree cover density |  | 0.16 ± 0.10 |  |  |  | 0.17 ± 0.11 |  |  |  | 0.13 ± 0.10 |  |  |  | 0.13 ± 0.11 |  |
| Soil-adjusted vegetation index |  | 0.42 ± 0.09 |  |  |  | 0.41 ± 0.08 |  |  |  | 0.38 ± 0.06 |  |  |  | 0.36 ± 0.06 |  |
| Green land use mix |  | 0.32 ± 0.11 |  |  |  | 0.31 ± 0.10 |  |  |  | 0.32 ± 0.11 |  |  |  | 0.30 ± 0.09 |  |
| **Outcomes** | | | | | | | | | | | | | | | |
| MET-minutes per week |  | 4038 (1906-6453) |  |  |  | 3234 (1526-5964) |  |  |  | 3150 (1413-7200) |  |  |  | 3610 (1482-6723) |  |
| Sitting time per day |  | 360 (240-520) |  |  |  | 420 (240-540) |  |  |  | 300 (210-480) |  |  |  | 360 (233-480) |  |

| **Table S4.** Description of participants’ individual and environmental characteristics by lifestyle preference (n=628) | | | | | | | | | | | | | | | |
| --- | --- | --- | --- | --- | --- | --- | --- | --- | --- | --- | --- | --- | --- | --- | --- |
| Variables | Mean ± SD or Proportion (%) | | | | | | | | | | | | | | |
|  | MET’HOOD 1 | | | | | | |  | MET’HOOD 2 | | | | | | |
|  | n |  | *# missing* |  | n |  | *# missing* |  | n |  | *# missing* |  | n |  | *# missing* |
|  |  |  |  |  |  |  |  |  |  |  |  |  |  |  |  |
|  | *Little to no importance*  *(n = 110)* | | |  | *Important (n = 518)* | | |  | *Little to no importance*  *(n = 92)* | | |  | *Important (n = 536)* | | |
| **Individual-level characteristics** | | | | | | | | | | | | | | | |
| **Age** |  | **46.1 ± 10.5** |  |  |  | **43.6 ± 12.2** |  |  |  | **52.9 ± 11.6** |  |  |  | **51.8 ± 11.9** |  |
| **Sex** |  |  |  |  |  |  |  |  |  |  |  |  |  |  |  |
| Men | 56 | 50.91 |  |  | 263 | 50.77 |  |  | 50 | 54.35 |  |  | 269 | 50.19 |  |
| Women | 54 | 49.09 |  |  | 255 | 49.23 |  |  | 42 | 45.65 |  |  | 267 | 49.81 |  |
| **Marital status** |  |  |  |  |  |  |  |  |  |  |  |  |  |  |  |
| Married / Living with partner | 86 | 78.18 |  |  | 382 | 73.75 |  |  | 73 | 79.34 |  |  | 414 | 77.24 |  |
| Single | 13 | 11.82 |  |  | 87 | 16.80 |  |  | 11 | 11.96 |  |  | 51 | 9.51 |  |
| Divorced / Separated / Widowed | 11 | 10.00 |  |  | 49 | 9.46 |  |  | 8 | 8.70 |  |  | 71 | 13.25 |  |
| **Education** |  |  | **1** |  |  |  | **3** |  |  |  | **1** |  |  |  | **4** |
| No diploma | 19 | 17.27 |  |  | 88 | 16.99 |  |  | 23 | 25.00 |  |  | 58 | 10.82 |  |
| Secondary level | 61 | 55.45 |  |  | 244 | 47.10 |  |  | 40 | 43.48 |  |  | 250 | 46.64 |  |
| University level | 29 | 26.36 |  |  | 183 | 35.33 |  |  | 28 | 30.43 |  |  | 224 | 41.79 |  |
| **Season (questionnaire completion date)** |  |  |  |  |  |  |  |  |  |  |  |  |  |  |  |
| Spring (March, April, May) | 26 | 23.63 |  |  | 145 | 27.99 |  |  | 26 | 28.26 |  |  | 154 | 28.73 |  |
| Summer (June, July, August) | 21 | 19.09 |  |  | 82 | 15.83 |  |  | 29 | 31.52 |  |  | 155 | 28.92 |  |
| Autumn (September, October, November) | 25 | 22.73 |  |  | 125 | 24.13 |  |  | 16 | 17.39 |  |  | 95 | 17.72 |  |
| Winter (December, January, February) | 38 | 34.55 |  |  | 166 | 32.05 |  |  | 21 | 22.83 |  |  | 132 | 24.63 |  |
| **Environmental-level characteristics** | | | | | | | | | | | | | | | |
| **Average housing price (sq m)** |  | **3475.03 ± 468.06** |  |  |  | **3569.00 ± 517.70** |  |  |  | **4808.71 ± 1143.47** |  |  |  | **5019.97 ± 1101.05** |  |
| **Building density** |  |  |  |  |  |  |  |  |  |  |  |  |  |  |  |
| Residential buildings | 628 | 0.066 ± 0.050 |  |  | 628 | 0.074 ± 0.050 |  |  | 628 | 0.065 ± 0.039 |  |  | 628 | 0.074 ± 0.049 |  |
| Non-residential buildings | 628 | 0.023 ± 0.017 |  |  | 628 | 0.025 ± 0.019 |  |  | 628 | 0.026 ± 0.022 |  |  | 628 | 0.029 ± 0.023 |  |
| **Greenness** |  |  |  |  |  |  |  |  |  |  |  |  |  |  |  |
| Tree cover density | 628 | 0.16 ± 0.10 |  |  | 628 | 0.17 ± 0.11 |  |  | 628 | 0.17 ± 0.13 |  |  | 628 | 0.13 ± 0.10 |  |
| Soil-adjusted vegetation index | 628 | 0.43 ± 0.08 |  |  | 628 | 0.41 ± 0.09 |  |  | 628 | 0.39 ± 0.06 |  |  | 628 | 0.37 ± 0.06 |  |
| Green land use mix | 628 | 0.33 ± 0.10 |  |  | 628 | 0.31 ± 0.10 |  |  | 628 | 0.34 ± 0.09 |  |  | 628 | 0.31 ± 0.10 |  |
| **Outcomes** | | | | | | | | | | | | | | | |
| MET-minutes per week | 104 | 3067 (1148 – 5339) | 6 |  | 494 | 3840 (1880 – 6458) | 24 |  | 46 | 1339 (396 – 4729) | 46 |  | 335 | 3612 (1644 – 7173) | 201 |
| Sitting time per day |  | 360 (300 – 600) | 1 |  |  | 360 (240 – 540) | 4 |  |  | 315 (240 – 480) | 24 |  |  | 330 (210 – 480) | 101 |

| **Table S5.** Description of participants’ individual and environmental characteristics by relocation status (n=628) | | | | | | | | | | | | | | | |
| --- | --- | --- | --- | --- | --- | --- | --- | --- | --- | --- | --- | --- | --- | --- | --- |
| Variables | Mean ± SD or Proportion (%) | | | | | | | | | | | | | | |
|  | MET’HOOD 1 | | | | | | |  | MET’HOOD 2 | | | | | | |
|  | n |  | *# missing* |  | n |  | *# missing* |  | n |  | *# missing* |  | n |  | *# missing* |
|  |  |  |  |  |  |  |  |  |  |  |  |  |  |  |  |
|  | *Non-mover (n = 433)* | | |  | *Relocated (n = 195)* | | |  | *Non-mover (n = 433)* | | |  | *Relocated (n = 195)* | | |
| **Individual-level characteristics** | | | | | | | | | | | | | | | |
| **Age** |  | **47.4 ± 10.8** |  |  |  | **36.5 ± 10.9** |  |  |  | **55.2 ± 10.8** |  |  |  | **44.7 ± 10.9** |  |
| **Sex** | **433** |  |  |  | **195** |  |  |  | **433** |  |  |  | **195** |  |  |
| Men | 209 | 48.27 |  |  | 110 | 56.41 |  |  | 209 | 48.27 |  |  | 110 | 56.41 |  |
| Women | 224 | 51.73 |  |  | 85 | 43.59 |  |  | 224 | 51.73 |  |  | 85 | 43.59 |  |
| **Marital status** | **433** |  |  |  | **195** |  |  |  | **433** |  |  |  | **195** |  |  |
| Married / Living with partner | 348 | 80.37 |  |  | 120 | 61.54 |  |  | 349 | 80.60 |  |  | 138 | 70.77 |  |
| Single | 43 | 9.93 |  |  | 57 | 29.23 |  |  | 34 | 7.85 |  |  | 28 | 14.36 |  |
| Divorced / Separated / Widowed | 42 | 9.70 |  |  | 18 | 9.23 |  |  | 50 | 11.55 |  |  | 29 | 14.87 |  |
| **Education** | **430** |  | **3** |  | **194** |  | **1** |  | **429** |  | **4** |  | **194** |  | **1** |
| No diploma | 71 | 16.51 |  |  | 36 | 18.56 |  |  | 53 | 12.35 |  |  | 28 | 14.43 |  |
| Secondary level | 224 | 52.09 |  |  | 81 | 41.75 |  |  | 213 | 49.65 |  |  | 77 | 39.69 |  |
| University level | 135 | 31.40 |  |  | 77 | 39.69 |  |  | 163 | 38.00 |  |  | 89 | 45.88 |  |
| **Lifestyle preference - Importance of PA** | **433** |  |  |  | **195** |  |  |  | **433** |  |  |  | **195** |  |  |
| Important | 354 | 81.76 |  |  | 164 | 84.10 |  |  | 368 | 84.99 |  |  | 168 | 86.15 |  |
| Little to no importance | 79 | 18.24 |  |  | 31 | 15.90 |  |  | 65 | 15.01 |  |  | 27 | 13.85 |  |
| **Season (questionnaire completion date)** | **433** |  |  |  | **195** |  |  |  | **433** |  |  |  | **195** |  |  |
| Spring (March, April, May) | 114 | 26.33 |  |  | 57 | 29.23 |  |  | 159 | 36.72 |  |  | 21 | 10.77 |  |
| Summer (June, July, August) | 71 | 16.40 |  |  | 32 | 16.41 |  |  | 104 | 24.02 |  |  | 80 | 41.03 |  |
| Autumn (September, October, November) | 103 | 23.79 |  |  | 47 | 24.10 |  |  | 57 | 13.16 |  |  | 54 | 27.69 |  |
| Winter (December, January, February) | 145 | 33.49 |  |  | 59 | 30.26 |  |  | 113 | 26.10 |  |  | 40 | 20.51 |  |
| **Environmental-level characteristics** | | | | | | | | | | | | | | | |
| **Relocation status** |  |  |  |  |  |  |  |  |  |  |  |  |  |  |  |
| Non mover |  |  |  |  |  |  |  |  |  |  |  |  |  |  |  |
| Relocated |  |  |  |  |  |  |  |  |  |  |  |  |  |  |  |
| **Average housing price (sq m)** |  | **3455 (3208-3905)** |  |  |  | **3531 (3221-4150)** |  |  |  | **4614 (4298-5707)** |  |  |  | **4778 (4298-5776)** |  |
| **Building density** |  |  |  |  |  |  |  |  |  |  |  |  |  |  |  |
| Residential buildings | 628 | 0.067 ± 0.045 |  |  | 628 | 0.085 ± 0.058 |  |  | 628 | 0.072 ± 0.046 |  |  | 628 | 0.075 ± 0.051 |  |
| Non-residential buildings | 628 | 0.023 ± 0.018 |  |  | 628 | 0.028 ± 0.020 |  |  | 628 | 0.027 ± 0.022 |  |  | 628 | 0.030 ± 0.024 |  |
| **Greenness** |  |  |  |  |  |  |  |  |  |  |  |  |  |  |  |
| Tree cover density | 628 | 0.17 ± 0.11 |  |  | 628 | 0.16 ± 0.10 |  |  | 628 | 0.13 ± 0.10 |  |  | 628 | 0.13 ± 0.11 |  |
| Soil-adjusted vegetation index | 628 | 0.42 ± 0.08 |  |  | 628 | 0.40 ± 0.09 |  |  | 628 | 0.37 ± 0.06 |  |  | 628 | 0.36 ± 0.06 |  |
| Green land use mix | 628 | 0.32 ± 0.10 |  |  | 628 | 0.29 ± 0.11 |  |  | 628 | 0.32 ± 0.10 |  |  | 628 | 0.30 ± 0.10 |  |
| **Outcomes** | | | | | | | | | | | | | | | |
| MET-minutes per week |  | 3861 (1869-6933) |  |  |  | 3280 (1494-5157) |  |  |  | 3704 (1516-7480) |  |  |  | 2853 (1254-5792) |  |
| Sitting time per day |  | 360 (240-510) |  |  |  | 360 (240-600) |  |  |  | 300 (240-480) |  |  |  | 360 (180-480) |  |

**Greenness exposure and building density for all buffers sizes**

| Table S6. Greenness exposure and building density in all buffer sizes (500, 800, 1000m) (n=628) | | | | | | |
| --- | --- | --- | --- | --- | --- | --- |
| **Buffer size (m)** | 500 | | 800 | | 1000 | |
|  | Mean ± SD | Range | Mean ± SD | Range | Mean ± SD | Range |
| **Wave 1** |  |  |  |  |  |  |
| **Greenness (0 – 1)** |  |  |  |  |  |  |
| TCD | 0.14 ± 0.11 | 0.00 – 0.63 | 0.16 ± 0.11 | 0.0031 – 0.59 | 0.17 ± 0.11 | 0.0027 – 0.59 |
| SAVI | 0.39 ± 0.35 | 0.12 – 0.61 | 0.41 ± 0.09 | 0.17 – 0.59 | 0.42 ± 0.09 | 0.18 – 0.59 |
| GLUM | 0.24 ± 0.10 | 0.01 – 0.49 | 0.29 ± 0.10 | 0.04 – 0.55 | 0.31 ± 0.10 | 0.059 – 0.55 |
| **Building density (0 – 1)** |  |  |  |  |  |  |
| Residential buildings | 0.096 ± 0.061 | 0.00088 – 0.41 | 0.081 ± 0.054 | 0.00053 – 0.31 | 0.072 ± 0.050 | 0.0010 – 0.26 |
| Nonresidential buildings | 0.027 ± 0.023 | 0.00 – 0.16 | 0.026 ± 0.019 | 0.00014 – 0.11 | 0.025 ± 0.019 | 0.0013 – 0.10 |
| **Wave 2** |  |  |  |  |  |  |
| **Greenness (0 – 1)** |  |  |  |  |  |  |
| TCD | 0.10 ± 0.10 | 0.00 – 0.57 | 0.12 ± 0.10 | 0.0064 – 0.56 | 0.13 ± 0.11 | 0.0058 – 0.57 |
| SAVI | 0.35 ± 0.06 | 0.13 – 0.53 | 0.36 ± 0.06 | 0.14 – 0.52 | 0.37 ± 0.06 | 0.17 – 0.52 |
| GLUM | 0.24 ± 0.10 | 0.0080 – 0.50 | 0.28 ± 0.10 | 0.019 – 0.56 | 0.31 ± 0.10 | 0.055 – 0.54 |
| **Building density (0 – 1)** |  |  |  |  |  |  |
| Residential buildings | 0.097 ± 0.057 | 0.00038 – 0.38 | 0.082 ± 0.051 | 0.00065 – 0.30 | 0.073 ± 0.048 | 0.00086 – 0.26 |
| Nonresidential buildings | 0.030 ± 0.026 | 0.00 – 0.20 | 0.029 ± 0.023 | 0.00033 – 0.14 | 0.028 ± 0.023 | 0.00028 – 0.12 |
| TCD = Tree Cover Density; SAVI = Soil-Adjusted Vegetation Index; GLUM = Green Land Use Mix.  All measures are given on a scale from 0-1. with higher values indicating a greater percentage of the participant’s buffer area being covered by the exposure measure. | | | | | | |
