# Supplementary material for: Longitudinal study of changes in greenness exposure, physical activity and sedentary behavior in the ORISCAV-LUX cohort study
Source: Int J Health Geogr. 2024 May 21;23:14. doi: 10.1186/s12942-024-00374-7 (PMC11110334; doi:10.1186/s12942-024-00374-7)
Supplement: Supplementary file 3 — Supplementary Material 3: Table S1. Results sensitivity analyses for greenness exposure and PA including interactions with sex (500m, 800m buffers). Table S2. Results sensitivity analyses for greenness exposure and SB including interactions with sex (500m, 800m buffers). Table S3. Results sensitivity analyses for greenness exposure and PA including interactions with NSES (500m, 800m buffers). Table S4. Results sensitivity analyses for greenness exposure and SB including interactions with NSES (500m, 800m buffers). Table S5. Results sensitivity analyses for greenness exposure and PA including interactions with lifestyle preference [for being active] (500m, 800m buffers). Table S6. Results sensitivity analyses for greenness exposure and SB including interactions with lifestyle preference [for being active] (500m, 800m buffers). Table S7. Results sensitivity analyses for greenness exposure and PA including interactions with relocation status (1000m buffer). Table S8. Results sensitivity analyses for greenness exposure and SB including interactions with relocation status (1000m buffer). Table S9. Results sensitivity analyses for greenness exposure and PA including interactions with relocation status (500m, 800m buffer). Table S10. Results sensitivity analyses for greenness exposure and SB including interactions with relocation status (500m, 800m buffer). Figure S1. Predicted PA and SB values by sex in the 800m buffer. Figure S2. Predicted SB values by neighborhood socio-economic status in the 500m buffer. Figure S3. Predicted PA and SB values by lifestyle preference [for being active] in the 500m buffer. Figure S4. Predicted SB values by lifestyle preference [for being active] in the 800m buffer. Figure S5. Predicted PA and SB values by relocation status in the 500m buffer. Figure S6. Predicted SB values by relocation status in the 800m buffer. [file 12942_2024_374_MOESM3_ESM.docx]

**Additional file 3**

| Table S1. Results sensitivity analyses for greenness exposure and PA including interactions with sex (500m, 800m buffers). | | | | | | | | | | | | | | | | | | | | | | | |
| --- | --- | --- | --- | --- | --- | --- | --- | --- | --- | --- | --- | --- | --- | --- | --- | --- | --- | --- | --- | --- | --- | --- | --- |
|  | **500 meter** | | | | | | | | | | |  | **800 meter** | | | | | | | | | | |
|  | **Model 1** | | | | |  | **Model 2** | | | | |  | **Model 1** | | | | |  | **Model 2** | | | | |
|  | β | exp(β) | 95% CI | | p |  | β | exp(β) | 95% CI | | p |  | β | exp(β) | 95% CI | | p |  | β | exp(β) | 95% CI | | p |
| **Physical Activity** | | | | | | | | | | | | | | | | | | | | | | | |
| **TCD** |  |  |  |  |  |  |  |  |  |  |  |  |  |  |  |  |  |  |  |  |  |  |  |
| **Between effect** | -0.72 | 0.49 | -1.89 | 0.46 | 0.23 |  | -1.46 | 0.23 | -3.20 | 0.28 | 0.10 |  | -0.44 | 0.64 | -1.51 | 0.62 | 0.41 |  | -1.29 | 0.27 | -2.86 | 0.27 | 0.11 |
| Female |  |  |  |  |  |  | -0.07 | 0.93 | -0.40 | 0.25 | 0.65 |  |  |  |  |  |  |  | -0.14 | 0.87 | -0.48 | 0.20 | 0.43 |
| TCD*Female |  |  |  |  |  |  | 1.35 | 3.86 | -0.75 | 3.45 | 0.21 |  |  |  |  |  |  |  | 1.60 | 4.96 | -0.40 | 3.61 | 0.12 |
| **Within effect** | **-2.06** | **0.13** | **-4.06** | **-0.06** | **0.04** |  | **-3.13** | **0.04** | **-5.62** | **-0.64** | **0.01** |  | **-2.25** | **0.11** | **-4.38** | **-0.11** | **0.04** |  | **-3.67** | **0.03** | **-6.34** | **-1.01** | **0.01** |
| Female |  |  |  |  |  |  | -0.07 | 0.93 | -0.40 | 0.25 | 0.65 |  |  |  |  |  |  |  | -0.14 | 0.87 | -0.48 | 0.20 | 0.43 |
| TCD*Female |  |  |  |  |  |  | 2.79 | 16.27 | -0.86 | 6.43 | 0.13 |  |  |  |  |  |  |  | **3.67** | **39.21** | **-0.26** | **7.60** | **0.07** |
| **SAVI** |  |  |  |  |  |  |  |  |  |  |  |  |  |  |  |  |  |  |  |  |  |  |  |
| **Between effect** | -1.69 | 0.19 | -4.67 | 1.30 | 0.27 |  | -0.50 | 0.61 | -4.04 | 3.04 | 0.78 |  | -1.40 | 0.25 | -4.66 | 1.87 | 0.40 |  | -0.43 | 0.65 | -4.22 | 3.37 | 0.83 |
| Female |  |  |  |  |  |  | 0.95 | 0.95 | -0.18 | 2.07 | 0.10 |  |  |  |  |  |  |  | 0.81 | 2.25 | -0.43 | 2.04 | 0.20 |
| SAVI*Female |  |  |  |  |  |  | -2.27 | 0.10 | -5.19 | 0.65 | 0.13 |  |  |  |  |  |  |  | -1.87 | 0.15 | -4.97 | 1.24 | 0.24 |
| **Within effect** | -0.65 | 0.52 | -4.12 | 2.81 | 0.71 |  | -1.10 | 0.33 | -5.24 | 3.04 | 0.60 |  | -0.16 | 0.85 | -3.81 | 3.49 | 0.93 |  | -0.86 | 0.42 | -5.15 | 3.43 | 0.69 |
| Female |  |  |  |  |  |  | 0.95 | 0.95 | -0.18 | 2.07 | 0.10 |  |  |  |  |  |  |  | 0.81 | 2.25 | -0.43 | 2.04 | 0.20 |
| SAVI*Female |  |  |  |  |  |  | 1.00 | 2.73 | -3.51 | 5.51 | 0.66 |  |  |  |  |  |  |  | 1.48 | 4.40 | -2.93 | 5.89 | 0.51 |
| **GLUM** |  |  |  |  |  |  |  |  |  |  |  |  |  |  |  |  |  |  |  |  |  |  |  |
| **Between effects** | -1.64 | 0.19 | -3.37 | 0.09 | 0.06 |  | -1.09 | 0.34 | -3.08 | 0.91 | 0.28 |  | **-1.79** | **0.17** | **-3.48** | **-0.11** | **0.04** |  | -1.16 | 0.31 | -3.11 | 0.78 | 0.24 |
| Female |  |  |  |  |  |  | 0.39 | 1.48 | -0.14 | 0.93 | 0.15 |  |  |  |  |  |  |  | 0.50 | 1.65 | -0.12 | 1.12 | 0.12 |
| GLUM*Female |  |  |  |  |  |  | -1.25 | 0.29 | -3.28 | 0.78 | 0.23 |  |  |  |  |  |  |  | -1.43 | 0.24 | -3.44 | 0.58 | 0.16 |
| **Within effect** | -2.17 | 0.11 | -5.83 | 1.48 | 0.24 |  | -0.97 | 0.38 | -5.26 | 3.32 | 0.66 |  | 0.08 | 1.08 | -3.69 | 3.85 | 0.97 |  | 1.01 | 2.75 | -3.30 | 5.32 | 0.65 |
| Female |  |  |  |  |  |  | 0.39 | 1.48 | -0.14 | 0.93 | 0.15 |  |  |  |  |  |  |  | 0.50 | 1.65 | -0.12 | 1.12 | 0.12 |
| GLUM*Female |  |  |  |  |  |  | -2.67 | 0.07 | -7.78 | 2.44 | 0.30 |  |  |  |  |  |  |  | -2.42 | 0.09 | -7.93 | 3.09 | 0.39 |
| Log-transformed coefficients are displayed. PA is given in MET-minutes per week. Between- and within-effects refer to the between- and within-component of the exposure measure and were assessed within the same model. We ran separate models for each exposure measure, i.e. TCD = Tree Cover Density; SAVI = Soil-Adjusted Vegetation Index; GLUM = Green Land Use Mix. Significant results are displayed **in bold. Model 1** adjusted for: sex, lifestyle preference, age, education, marital status, housing price, nonresidential and residential building density, date of questionnaire completion, relocation status. **Model 2** adjusted for: sex, lifestyle preference, age, education, marital status, housing price, nonresidential and residential building density, date of questionnaire completion, relocation status and interaction term with sex. | | | | | | | | | | | | | | | | | | | | | | | |

| Table S2. Results sensitivity analyses for greenness exposure and SB including interactions with sex (500m, 800m buffers). | | | | | | | | | | | | | | | | | | | |
| --- | --- | --- | --- | --- | --- | --- | --- | --- | --- | --- | --- | --- | --- | --- | --- | --- | --- | --- | --- |
|  | **500 meter** | | | | | | | | |  | **800 meter** | | | | | | | | |
|  | **Model 1** | | | |  | **Model 2** | | | |  | **Model 1** | | | |  | **Model 2** | | | |
|  | β | 95% CI | | p |  | β | 95% CI | | p |  | β | 95% CI | | p |  | β | 95% CI | | p |
| **Sedentary Behavior** | | | | | | | | | | | | | | | | | | | |
| **TCD** |  |  |  |  |  |  |  |  |  |  |  |  |  |  |  |  |  |  |  |
| **Between effect** | 34.24 | -100.36 | 168.84 | 0.62 |  | 1.69 | -190.40 | 193.78 | 0.99 |  | 5.19 | -121.21 | 131.59 | 0.94 |  | -93.71 | -270.46 | 83.04 | 0.30 |
| Female |  |  |  |  |  | **-58.98** | **-98.07** | **-19.89** | **0.00** |  |  |  |  |  |  | **-77.54** | **-120.89** | **-34.19** | **0.00** |
| TCD*Female |  |  |  |  |  | 58.87 | -194.92 | 312.66 | 0.65 |  |  |  |  |  |  | 176.16 | -65.55 | 417.87 | 0.15 |
| **Within effect** | -2.34 | -199.56 | 194.88 | 0.98 |  | 41.47 | -199.35 | 282.30 | 0.74 |  | -32.96 | -248.42 | 182.51 | 0.76 |  | 122.36 | -151.45 | 396.18 | 0.38 |
| Female |  |  |  |  |  | **-58.98** | **-98.07** | **-19.89** | **0.00** |  |  |  |  |  |  | **-77.54** | **-120.89** | **-34.19** | **0.00** |
| TCD*Female |  |  |  |  |  | -114.91 | -469.31 | 239.49 | 0.52 |  |  |  |  |  |  | **-418.95** | **-808.24** | **-29.65** | **0.03** |
| **SAVI** |  |  |  |  |  |  |  |  |  |  |  |  |  |  |  |  |  |  |  |
| **Between effect** | 78.68 | -281.79 | 439.15 | 0.67 |  | 132.65 | -269.37 | 534.68 | 0.52 |  | 187.86 | -216.65 | 592.37 | 0.36 |  | 245.35 | -195.93 | 686.63 | 0.28 |
| Female |  |  |  |  |  | -13.16 | -142.01 | 115.69 | 0.84 |  |  |  |  |  |  | -7.60 | -143.66 | 128.47 | 0.91 |
| TCD*Female |  |  |  |  |  | -103.09 | -441.82 | 235.63 | 0.55 |  |  |  |  |  |  | -111.45 | -458.77 | 235.87 | 0.53 |
| **Within effect** | 38.72 | -304.69 | 382.13 | 0.82 |  | 77.72 | -311.91 | 467.36 | 0.70 |  | 7.46 | -340.97 | 355.89 | 0.97 |  | 61.38 | -339.00 | 461.76 | 0.76 |
| Female |  |  |  |  |  | -13.16 | -142.01 | 115.69 | 0.84 |  |  |  |  |  |  | -7.60 | -143.66 | 128.47 | 0.91 |
| TCD*Female |  |  |  |  |  | -81.98 | -487.31 | 323.36 | 0.69 |  |  |  |  |  |  | -109.74 | -519.23 | 299.74 | 0.60 |
| **GLUM** |  |  |  |  |  |  |  |  |  |  |  |  |  |  |  |  |  |  |  |
| **Between effects** | 20.74 | -181.47 | 222.94 | 0.84 |  | 91.28 | -139.46 | 322.03 | 0.44 |  | 138.30 | -67.82 | 344.43 | 0.19 |  | 190.11 | -41.62 | 421.85 | 0.11 |
| Female |  |  |  |  |  | -14.20 | -78.23 | 49.83 | 0.66 |  |  |  |  |  |  | -16.93 | -89.87 | 56.02 | 0.65 |
| TCD*Female |  |  |  |  |  | -159.41 | -410.82 | 92.00 | 0.21 |  |  |  |  |  |  | -118.48 | -360.58 | 123.61 | 0.34 |
| **Within effect** | 139.48 | -224.00 | 502.97 | 0.45 |  | 44.44 | -384.29 | 473.17 | 0.84 |  | 19.00 | -359.26 | 397.26 | 0.92 |  | -10.69 | -441.48 | 420.10 | 0.96 |
| Female |  |  |  |  |  | -14.20 | -78.23 | 49.83 | 0.66 |  |  |  |  |  |  | -16.93 | -89.87 | 56.02 | 0.65 |
| TCD*Female |  |  |  |  |  | 209.65 | -280.92 | 700.21 | 0.40 |  |  |  |  |  |  | 77.88 | -432.81 | 588.56 | 0.76 |
| SB is given as sitting time in minutes per day. Between- and within-effects refer to the between- and within-component of the exposure measure and were assessed within the same model. We ran separate models for each exposure measure, i.e. TCD = Tree Cover Density; SAVI = Soil-Adjusted Vegetation Index; GLUM = Green Land Use Mix. Significant results are displayed **in bold. Model 1** adjusted for: sex, lifestyle preference, age, education, marital status, housing price, nonresidential and residential building density, date of questionnaire completion, relocation status. **Model 2** adjusted for: sex, lifestyle preference, age, education, marital status, housing price, nonresidential and residential building density, date of questionnaire completion, relocation status and interaction term with sex. | | | | | | | | | | | | | | | | | | | |

| Table S3. Results sensitivity analyses for greenness exposure and PA including interactions with NSES (500m, 800m buffers). | | | | | | | | | | | | | | | | | | | | | | | |
| --- | --- | --- | --- | --- | --- | --- | --- | --- | --- | --- | --- | --- | --- | --- | --- | --- | --- | --- | --- | --- | --- | --- | --- |
|  | **500 meter** | | | | | | | | | | |  | **800 meter** | | | | | | | | | | |
|  | **Model 1** | | | | |  | **Model 2** | | | | |  | **Model 1** | | | | |  | **Model 2** | | | | |
|  | β | exp(β) | 95% CI | | p |  | β | exp(β) | 95% CI | | p |  | β | exp(β) | 95% CI | | p |  | β | exp(β) | 95% CI | | p |
| **Physical Activity** | | | | | | | | | | | | | | | | | | | | | | | |
| **TCD** |  |  |  |  |  |  |  |  |  |  |  |  |  |  |  |  |  |  |  |  |  |  |  |
| **Between effect** | -0.72 | -1.89 | 0.46 | 0.23 | -0.72 |  | -0.36 | 0.70 | -5.97 | 5.25 | 0.90 |  | -0.44 | -1.51 | 0.62 | 0.41 | -0.44 |  | -0.80 | 0.45 | -6.13 | 4.52 | 0.77 |
| Housing Price |  |  |  |  |  |  | 0.00 | 1.00 | 0.00 | 0.00 | 0.56 |  |  |  |  |  |  |  | 0.00 | 1.00 | 0.00 | 0.00 | 0.42 |
| TCD*Housing price |  |  |  |  |  |  | 0.00 | 1.00 | 0.00 | 0.00 | 0.90 |  |  |  |  |  |  |  | 0.00 | 1.00 | 0.00 | 0.00 | 0.89 |
| **Within effect** | **-2.06** | **-4.06** | **-0.06** | **0.04** | **-2.06** |  | -1.98 | 0.14 | -4.02 | 0.06 | 0.06 |  | **-2.25** | **-4.38** | **-0.11** | **0.04** | **-2.25** |  | -2.18 | 0.11 | -4.35 | -0.01 | 0.05 |
| Housing price change |  |  |  |  |  |  | 0.00 | 1.00 | 0.00 | 0.00 | 0.75 |  |  |  |  |  |  |  | 0.00 | 1.00 | 0.00 | 0.00 | 0.69 |
| TCD*Housing price change |  |  |  |  |  |  | 0.00 | 1.00 | 0.00 | 0.00 | 0.57 |  |  |  |  |  |  |  | 0.00 | 1.00 | 0.00 | 0.00 | 0.63 |
| **SAVI** |  |  |  |  |  |  |  |  |  |  |  |  |  |  |  |  |  |  |  |  |  |  |  |
| **Between effect** | -1.69 | -4.67 | 1.30 | 0.27 | -1.69 |  | -2.54 | 0.08 | -10.41 | 5.33 | 0.53 |  | -1.40 | -4.66 | 1.87 | 0.40 | -1.40 |  | -3.50 | 0.03 | -11.51 | 4.52 | 0.39 |
| Housing price |  |  |  |  |  |  | 0.00 | 1.00 | 0.00 | 0.00 | 0.53 |  |  |  |  |  |  |  | 0.00 | 1.00 | 0.00 | 0.00 | 0.37 |
| SAVI*Housing price |  |  |  |  |  |  | 0.00 | 1.00 | 0.00 | 0.00 | 0.76 |  |  |  |  |  |  |  | 0.00 | 1.00 | 0.00 | 0.00 | 0.54 |
| **Within effect** | -0.65 | -4.12 | 2.81 | 0.71 | -0.65 |  | -0.54 | 0.58 | -4.02 | 2.93 | 0.76 |  | -0.16 | -3.81 | 3.49 | 0.93 | -0.16 |  | -0.07 | 0.93 | -3.73 | 3.59 | 0.97 |
| Housing price change |  |  |  |  |  |  | 0.00 | 1.00 | 0.00 | 0.00 | 0.81 |  |  |  |  |  |  |  | 0.00 | 1.00 | 0.00 | 0.00 | 0.71 |
| SAVI*Housing price change |  |  |  |  |  |  | 0.00 | 1.00 | 0.00 | 0.01 | 0.46 |  |  |  |  |  |  |  | 0.00 | 1.00 | 0.00 | 0.00 | 0.54 |
| **GLUM** |  |  |  |  |  |  |  |  |  |  |  |  |  |  |  |  |  |  |  |  |  |  |  |
| **Between effects** | -1.64 | -3.37 | 0.09 | 0.06 | -1.64 |  | -1.37 | 0.25 | -7.14 | 4.39 | 0.64 |  | **-1.79** | **-3.48** | **-0.11** | **0.04** | **-1.79** |  | -2.82 | 0.06 | -8.35 | 2.70 | 0.32 |
| Housing price |  |  |  |  |  |  | 0.00 | 1.00 | 0.00 | 0.00 | 0.63 |  |  |  |  |  |  |  | 0.00 | 1.00 | 0.00 | 0.00 | 0.41 |
| GLUM*Housing price |  |  |  |  |  |  | 0.00 | 1.00 | 0.00 | 0.00 | 0.92 |  |  |  |  |  |  |  | 0.00 | 1.00 | 0.00 | 0.00 | 0.69 |
| **Within effect** | -2.17 | -5.83 | 1.48 | 0.24 | -2.17 |  | -1.88 | 0.15 | -5.55 | 1.79 | 0.32 |  | 0.08 | -3.69 | 3.85 | 0.97 | 0.08 |  | 0.33 | 1.39 | -3.47 | 4.13 | 0.86 |
| Housing price change |  |  |  |  |  |  | 0.00 | 1.00 | 0.00 | 0.00 | 0.78 |  |  |  |  |  |  |  | 0.00 | 1.00 | 0.00 | 0.00 | 0.71 |
| GLUM*Housing price change |  |  |  |  |  |  | 0.00 | 1.00 | 0.00 | 0.01 | 0.14 |  |  |  |  |  |  |  | 0.00 | 1.00 | 0.00 | 0.01 | 0.16 |
| Log-transformed coefficients are displayed. PA is given in MET-minutes per week. Between- and within-effects refer to the between- and within-component of the exposure measure and were assessed within the same model. We ran separate models for each exposure measure, i.e. TCD = Tree Cover Density; SAVI = Soil-Adjusted Vegetation Index; GLUM = Green Land Use Mix. Significant results are displayed **in bold. Model 1** adjusted for: sex, lifestyle preference, age, education, marital status, housing price, nonresidential and residential building density, date of questionnaire completion, relocation status. **Model 2** adjusted for: sex, lifestyle preference, age, education, marital status, housing price, nonresidential and residential building density, date of questionnaire completion, relocation status and interaction term with neighborhood socio-economic status. | | | | | | | | | | | | | | | | | | | | | | | |

| Table S4. Results sensitivity analyses for greenness exposure and SB including interactions with NSES (500m, 800m buffers). | | | | | | | | | | | | | | | | | | | |
| --- | --- | --- | --- | --- | --- | --- | --- | --- | --- | --- | --- | --- | --- | --- | --- | --- | --- | --- | --- |
|  | **500 meter** | | | | | | | | |  | **800 meter** | | | | | | | | |
|  | **Model 1** | | | |  | **Model 2** | | | |  | **Model 1** | | | |  | **Model 2** | | | |
|  | β | 95% CI | | p |  | β | 95% CI | | p |  | β | 95% CI | | p |  | β | 95% CI | | p |
| **Sedentary Behavior** | | | | | | | | | | | | | | | | | | | |
| **TCD** |  |  |  |  |  |  |  |  |  |  |  |  |  |  |  |  |  |  |  |
| **Between effect** | 34.24 | -100.36 | 168.84 | 0.62 |  | 276.82 | -421.05 | 974.68 | 0.44 |  | 5.19 | -121.21 | 131.59 | 0.94 |  | 160.70 | -508.21 | 829.60 | 0.64 |
| Housing Price |  |  |  |  |  | 0.02 | -0.01 | 0.05 | 0.13 |  |  |  |  |  |  | 0.02 | -0.01 | 0.05 | 0.18 |
| TCD*Housing price |  |  |  |  |  | -0.06 | -0.21 | 0.10 | 0.49 |  |  |  |  |  |  | -0.04 | -0.19 | 0.12 | 0.64 |
| **Within effect** | -2.34 | -199.56 | 194.88 | 0.98 |  | -2.94 | -199.95 | 194.06 | 0.98 |  | -32.96 | -248.42 | 182.51 | 0.76 |  | -33.39 | -248.57 | 181.78 | 0.76 |
| Housing price change |  |  |  |  |  | 0.01 | -0.01 | 0.04 | 0.20 |  |  |  |  |  |  | 0.02 | -0.01 | 0.04 | 0.19 |
| TCD*Housing price change |  |  |  |  |  | 0.06 | -0.24 | 0.36 | 0.71 |  |  |  |  |  |  | 0.05 | -0.27 | 0.36 | 0.77 |
| **SAVI** |  |  |  |  |  |  |  |  |  |  |  |  |  |  |  |  |  |  |  |
| **Between effect** | 78.68 | -281.79 | 439.15 | 0.67 |  | -252.42 | -1160.73 | 655.90 | 0.59 |  | 187.86 | -216.65 | 592.37 | 0.36 |  | -162.66 | -1085.55 | 760.22 | 0.73 |
| Housing price |  |  |  |  |  | -0.01 | -0.09 | 0.06 | 0.77 |  |  |  |  |  |  | -0.02 | -0.09 | 0.06 | 0.69 |
| SAVI*Housing price |  |  |  |  |  | 0.07 | -0.14 | 0.27 | 0.52 |  |  |  |  |  |  | 0.08 | -0.13 | 0.28 | 0.47 |
| **Within effect** | 38.72 | -304.69 | 382.13 | 0.82 |  | 41.22 | -301.49 | 383.93 | 0.81 |  | 7.46 | -340.97 | 355.89 | 0.97 |  | 9.32 | -338.68 | 357.33 | 0.96 |
| Housing price change |  |  |  |  |  | 0.01 | -0.01 | 0.04 | 0.23 |  |  |  |  |  |  | 0.02 | -0.01 | 0.04 | 0.21 |
| SAVI*Housing price change |  |  |  |  |  | **-0.35** | **-0.75** | **0.05** | **0.08** |  |  |  |  |  |  | -0.31 | -0.71 | 0.09 | 0.13 |
| **GLUM** |  |  |  |  |  |  |  |  |  |  |  |  |  |  |  |  |  |  |  |
| **Between effects** | 20.74 | -181.47 | 222.94 | 0.84 |  | -157.39 | -851.83 | 537.05 | 0.66 |  | 138.30 | -67.82 | 344.43 | 0.19 |  | 29.16 | -638.23 | 696.54 | 0.93 |
| Housing price |  |  |  |  |  | 0.00 | -0.04 | 0.04 | 0.87 |  |  |  |  |  |  | 0.01 | -0.04 | 0.05 | 0.78 |
| GLUM*Housing price |  |  |  |  |  | 0.04 | -0.12 | 0.21 | 0.60 |  |  |  |  |  |  | 0.03 | -0.13 | 0.18 | 0.74 |
| **Within effect** | 139.48 | -224.00 | 502.97 | 0.45 |  | 142.26 | -222.20 | 506.73 | 0.44 |  | 139.48 | -224.00 | 502.97 | 0.45 |  | 20.48 | -358.27 | 399.22 | 0.92 |
| Housing price change |  |  |  |  |  | 0.02 | -0.01 | 0.04 | 0.20 |  |  |  |  |  |  | 0.02 | -0.01 | 0.04 | 0.19 |
| GLUM*Housing price change |  |  |  |  |  | -0.30 | -0.71 | 0.12 | 0.16 |  |  |  |  |  |  | -0.15 | -0.57 | 0.27 | 0.49 |
| SB is given as sitting time in minutes per day. Between- and within-effects refer to the between- and within-component of the exposure measure and were assessed within the same model. We ran separate models for each exposure measure, i.e. TCD = Tree Cover Density; SAVI = Soil-Adjusted Vegetation Index; GLUM = Green Land Use Mix. NSES = Neighborhood socio-economic status. Significant results are displayed **in bold. Model 1** adjusted for: sex, lifestyle preference, age, education, marital status, housing price, nonresidential and residential building density, date of questionnaire completion, relocation status. **Model 2** adjusted for: sex, lifestyle preference, age, education, marital status, housing price, nonresidential and residential building density, date of questionnaire completion, relocation status and interaction term with neighborhood socio-economic status. | | | | | | | | | | | | | | | | | | | |

| Table S5. Results sensitivity analyses for greenness exposure and PA including interactions with lifestyle preference [for being active] (500m, 800m buffers). | | | | | | | | | | | | | | | | | | | | | | | |
| --- | --- | --- | --- | --- | --- | --- | --- | --- | --- | --- | --- | --- | --- | --- | --- | --- | --- | --- | --- | --- | --- | --- | --- |
|  | **500 meter** | | | | | | | | | | |  | **800 meter** | | | | | | | | | | |
|  | **Model 1** | | | | |  | **Model 2** | | | | |  | **Model 1** | | | | |  | **Model 2** | | | | |
|  | β | exp(β) | 95% CI | | p |  | β | exp(β) | 95% CI | | p |  | β | exp(β) | 95% CI | | p |  | β | exp(β) | 95% CI | | p |
| **Physical Activity** | | | | | | | | | | | | | | | | | | | | | | | |
| **TCD** |  |  |  |  |  |  |  |  |  |  |  |  |  |  |  |  |  |  |  |  |  |  |  |
| **Between effect** | -0.72 | -1.89 | 0.46 | 0.23 | -0.72 |  | -0,47 | 0,62 | -1,73 | 0,78 | 0,46 |  | -0.44 | -1.51 | 0.62 | 0.41 | -0.44 |  | -0,26 | 0,77 | -1,42 | 0,89 | 0,65 |
| Lifestyle preference |  |  |  |  |  |  | -0,45 | 0,64 | -0,88 | -0,02 | **0,04** |  |  |  |  |  |  |  | -0,48 | 0,62 | -0,93 | -0,02 | **0,04** |
| TCD*Lifestyle preference |  |  |  |  |  |  | -1,30 | 0,27 | -3,97 | 1,37 | 0,34 |  |  |  |  |  |  |  | -0,91 | 0,40 | -3,44 | 1,63 | 0,48 |
| **Within effect** | **-2.06** | **-4.06** | **-0.06** | **0.04** | **-2.06** |  | -1,96 | 0,14 | -4,15 | 0,23 | 0,08 |  | **-2.25** | **-4.38** | **-0.11** | **0.04** | **-2.25** |  | -2,33 | 0,10 | -4,67 | 0,02 | 0,05 |
| Lifestyle preference |  |  |  |  |  |  | -0,45 | 0,64 | -0,88 | -0,02 | **0,04** |  |  |  |  |  |  |  | -0,48 | 0,62 | -0,93 | -0,02 | **0,04** |
| TCD*Lifestyle preference |  |  |  |  |  |  | -0,88 | 0,41 | -5,96 | 4,19 | 0,73 |  |  |  |  |  |  |  | 0,24 | 1,27 | -5,06 | 5,55 | 0,93 |
| **SAVI** |  |  |  |  |  |  |  |  |  |  |  |  |  |  |  |  |  |  |  |  |  |  |  |
| **Between effect** | -1.69 | -4.67 | 1.30 | 0.27 | -1.69 |  | -1,66 | 0,19 | -4,62 | 1,30 | 0,27 |  | -1.40 | -4.66 | 1.87 | 0.40 | -1.40 |  | -1,29 | 0,28 | -4,53 | 1,96 | 0,44 |
| Lifestyle preference |  |  |  |  |  |  | -0,57 | 0,56 | -2,20 | 1,06 | 0,49 |  |  |  |  |  |  |  | -0,29 | 0,75 | -2,05 | 1,47 | 0,75 |
| SAVI*Lifestyle preference |  |  |  |  |  |  | 0,04 | 1,04 | -4,26 | 4,34 | 0,99 |  |  |  |  |  |  |  | -0,70 | 0,49 | -5,19 | 3,78 | 0,76 |
| **Within effect** | -0.65 | -4.12 | 2.81 | 0.71 | -0.65 |  | 0,31 | 1,37 | -3,31 | 3,93 | 0,87 |  | -0.16 | -3.81 | 3.49 | 0.93 | -0.16 |  | 0,57 | 1,76 | -3,27 | 4,40 | 0,77 |
| Lifestyle preference |  |  |  |  |  |  | -0,57 | 0,56 | -2,20 | 1,06 | 0,49 |  |  |  |  |  |  |  | -0,29 | 0,75 | -2,05 | 1,47 | 0,75 |
| SAVI*Lifestyle preference |  |  |  |  |  |  | -4,84 | 0,01 | -10,72 | 1,05 | 0,11 |  |  |  |  |  |  |  | -3,83 | 0,02 | -9,86 | 2,21 | 0,21 |
| **GLUM** |  |  |  |  |  |  |  |  |  |  |  |  |  |  |  |  |  |  |  |  |  |  |  |
| **Between effects** | -1.64 | -3.37 | 0.09 | 0.06 | -1.64 |  | -1,84 | 0,16 | -3,59 | -0,09 | **0,04** |  | **-1.79** | **-3.48** | **-0.11** | **0.04** | **-1.79** |  | -1,97 | 0,14 | -3,70 | -0,24 | **0,03** |
| Lifestyle preference |  |  |  |  |  |  | -0,93 | 0,39 | -1,67 | -0,20 | **0,01** |  |  |  |  |  |  |  | -0,97 | 0,38 | -1,83 | -0,11 | **0,03** |
| GLUM*Lifestyle preference |  |  |  |  |  |  | 1,38 | 3,99 | -1,38 | 4,15 | 0,33 |  |  |  |  |  |  |  | 1,24 | 3,46 | -1,46 | 3,94 | 0,37 |
| **Within effect** | -2.17 | -5.83 | 1.48 | 0.24 | -2.17 |  | -1,23 | 0,29 | -4,87 | 2,41 | 0,51 |  | 0.08 | -3.69 | 3.85 | 0.97 | 0.08 |  | 0,48 | 1,62 | -3,29 | 4,26 | 0,80 |
| Lifestyle preference |  |  |  |  |  |  | -0,93 | 0,39 | -1,67 | -0,20 | **0,01** |  |  |  |  |  |  |  | -0,97 | 0,38 | -1,83 | -0,11 | **0,03** |
| GLUM* Lifestyle preference |  |  |  |  |  |  | -13,10 | 0,00 | -21,98 | -4,22 | **0,00** |  |  |  |  |  |  |  | -5,37 | 0,00 | -14,60 | 3,87 | 0,25 |
| Log-transformed coefficients are displayed. PA is given in MET-minutes per week. Between- and within-effects refer to the between- and within-component of the exposure measure and were assessed within the same model. We ran separate models for each exposure measure, i.e. TCD = Tree Cover Density; SAVI = Soil-Adjusted Vegetation Index; GLUM = Green Land Use Mix. Significant results are displayed **in bold. Model 1** adjusted for: sex, lifestyle preference, age, education, marital status, housing price, nonresidential and residential building density, date of questionnaire completion, relocation status. **Model 2** adjusted for: sex, lifestyle preference, age, education, marital status, housing price, nonresidential and residential building density, date of questionnaire completion, relocation status and interaction term with lifestyle preference [for being active]. | | | | | | | | | | | | | | | | | | | | | | | |

| Table S6. Results sensitivity analyses for greenness exposure and SB including interactions with lifestyle preference [for being active] (500m, 800m buffers). | | | | | | | | | | | | | | | | | | | |
| --- | --- | --- | --- | --- | --- | --- | --- | --- | --- | --- | --- | --- | --- | --- | --- | --- | --- | --- | --- |
|  | **500 meter** | | | | | | | | |  | **800 meter** | | | | | | | | |
|  | **Model 1** | | | |  | **Model 2** | | | |  | **Model 1** | | | |  | **Model 2** | | | |
|  | β | 95% CI | | p |  | β | 95% CI | | p |  | β | 95% CI | | p |  | β | 95% CI | | p |
| **Sedentary Behavior** | | | | | | | | | | | | | | | | | | | |
| **TCD** |  |  |  |  |  |  |  |  |  |  |  |  |  |  |  |  |  |  |  |
| **Between effect** | 34.24 | -100.36 | 168.84 | 0.62 |  | 71,23 | -73,19 | 215,66 | 0,33 |  | 5.19 | -121.21 | 131.59 | 0.94 |  | 35,74 | -100,60 | 172,08 | 0,61 |
| Lifestyle preference |  |  |  |  |  | 53,21 | 6,18 | 100,24 | 0,03 |  |  |  |  |  |  | 51,55 | 1,64 | 101,45 | 0,04 |
| TCD*Lifestyle preference |  |  |  |  |  | -182,10 | -481,04 | 116,84 | 0,23 |  |  |  |  |  |  | -148,49 | -434,04 | 137,07 | 0,31 |
| **Within effect** | -2.34 | -199.56 | 194.88 | 0.98 |  | 6,90 | -212,25 | 226,06 | 0,95 |  | -32.96 | -248.42 | 182.51 | 0.76 |  | -47,85 | -286,37 | 190,66 | 0,69 |
| Lifestyle preference |  |  |  |  |  | 53,21 | 6,18 | 100,24 | 0,03 |  |  |  |  |  |  | 51,55 | 1,64 | 101,45 | 0,04 |
| TCD*Lifestyle preference |  |  |  |  |  | -82,30 | -606,66 | 442,06 | 0,76 |  |  |  |  |  |  | 54,55 | -506,16 | 615,25 | 0,85 |
| **SAVI** |  |  |  |  |  |  |  |  |  |  |  |  |  |  |  |  |  |  |  |
| **Between effect** | 78.68 | -281.79 | 439.15 | 0.67 |  | 157,22 | -209,34 | 523,78 | 0,40 |  | 187.86 | -216.65 | 592.37 | 0.36 |  | 252,86 | -155,19 | 660,91 | 0,22 |
| Lifestyle preference |  |  |  |  |  | 189,38 | 27,23 | 351,52 | 0,02 |  |  |  |  |  |  | 198,09 | 22,78 | 373,40 | 0,03 |
| SAVI*Lifestyle preference |  |  |  |  |  | -416,81 | -830,77 | -2,85 | 0,05 |  |  |  |  |  |  | -427,41 | -863,44 | 8,61 | 0,05 |
| **Within effect** | 38.72 | -304.69 | 382.13 | 0.82 |  | 28,01 | -336,76 | 392,77 | 0,88 |  | 7.46 | -340.97 | 355.89 | 0.97 |  | -5,24 | -370,07 | 359,58 | 0,98 |
| Lifestyle preference |  |  |  |  |  | 189,38 | 27,23 | 351,52 | 0,02 |  |  |  |  |  |  | 198,09 | 22,78 | 373,40 | 0,03 |
| SAVI*Lifestyle preference |  |  |  |  |  | 109,81 | -478,60 | 698,22 | 0,71 |  |  |  |  |  |  | 144,87 | -462,69 | 752,42 | 0,64 |
| **GLUM** |  |  |  |  |  |  |  |  |  |  |  |  |  |  |  |  |  |  |  |
| **Between effects** | 20.74 | -181.47 | 222.94 | 0.84 |  | 54,91 | -152,54 | 262,37 | 0,60 |  | 138.30 | -67.82 | 344.43 | 0.19 |  | 168,31 | -42,53 | 379,15 | 0,12 |
| Lifestyle preference |  |  |  |  |  | 78,69 | -3,90 | 161,28 | 0,06 |  |  |  |  |  |  | 80,61 | -14,88 | 176,11 | 0,10 |
| GLUM*Lifestyle preference |  |  |  |  |  | -192,65 | -495,06 | 109,76 | 0,21 |  |  |  |  |  |  | -171,39 | -470,03 | 127,24 | 0,26 |
| **Within effect** | 139.48 | -224.00 | 502.97 | 0.45 |  | 154,25 | -214,32 | 522,82 | 0,41 |  | 139.48 | -224.00 | 502.97 | 0.45 |  | 60,53 | -321,32 | 442,39 | 0,76 |
| Lifestyle preference |  |  |  |  |  | 78,69 | -3,90 | 161,28 | 0,06 |  |  |  |  |  |  | 80,61 | -14,88 | 176,11 | 0,10 |
| GLUM* Lifestyle preference |  |  |  |  |  | -186,66 | -1087,23 | 713,91 | 0,68 |  |  |  |  |  |  | -494,45 | -1418,95 | 430,05 | 0,29 |
| SB is given as sitting time in minutes per day. Between- and within-effects refer to the between- and within-component of the exposure measure and were assessed within the same model. We ran separate models for each exposure measure, i.e. TCD = Tree Cover Density; SAVI = Soil-Adjusted Vegetation Index; GLUM = Green Land Use Mix. NSES = Neighborhood socio-economic status. Significant results are displayed **in bold. Model 1** adjusted for: sex, lifestyle preference, age, education, marital status, housing price, nonresidential and residential building density, date of questionnaire completion, relocation status. **Model 2** adjusted for: sex, lifestyle preference, age, education, marital status, housing price, nonresidential and residential building density, date of questionnaire completion, relocation status and interaction term with lifestyle preference [for being active]. | | | | | | | | | | | | | | | | | | | |

| Table S7. Results sensitivity analyses for greenness exposure and PA including interactions with relocation status (1000m buffer). | | | | | | | | | | | |
| --- | --- | --- | --- | --- | --- | --- | --- | --- | --- | --- | --- |
|  | Model 1 | | | | |  | Model 2 (relocation) | | | | |
|  | β | exp(β) | 95% CI | | P |  | β | exp(β) | 95% CI | | P |
| **Physical Activity** | | | | | | | | | | | |
| **TCD** |  |  |  |  |  |  |  |  |  |  |  |
| **Between effect** | -0.44 | 0.65 | -1.49 | 0.62 | 0.41 |  | -0.16 | 0.85 | -1.37 | 1.05 | 0.79 |
| Relocation |  |  |  |  |  |  | -0.03 | 0.97 | -0.46 | 0.39 | 0.88 |
| TCD*Relocation |  |  |  |  |  |  | -1.34 | 0.26 | -3.82 | 1.14 | 0.29 |
| **Within effect** | **-2.60** | **0.07** | **-4.75** | **-0.44** | **0.02** |  | -3.56 | 0.03 | -8.50 | 1.37 | 0.16 |
| Relocation |  |  |  |  |  |  | -0.03 | 0.97 | -0.46 | 0.39 | 0.88 |
| TCD*Relocation |  |  |  |  |  |  | 1.12 | 3.08 | -4.13 | 6.38 | 0.67 |
| **SAVI** |  |  |  |  |  |  |  |  |  |  |  |
| **Between effect** | -1.38 | 0.25 | -4.76 | 2.00 | 0.42 |  | -1.47 | 0.23 | -4.93 | 1.99 | 0.40 |
| Relocation |  |  |  |  |  |  | -0.39 | 0.68 | -1.69 | 0.91 | 0.56 |
| SAVI*Relocation |  |  |  |  |  |  | 0.41 | 1.50 | -2.93 | 3.75 | 0.81 |
| **Within effect** | 0.16 | 1.17 | -3.69 | 4.00 | 0.94 |  | 0.78 | 2.18 | -3.72 | 5.28 | 0.73 |
| Relocation |  |  |  |  |  |  | -0.39 | 0.68 | -1.69 | 0.91 | 0.56 |
| SAVI*Relocation |  |  |  |  |  |  | -1.43 | 0.24 | -6.99 | 4.13 | 0.61 |
| **GLUM** |  |  |  |  |  |  |  |  |  |  |  |
| **Between effect** | **-2.02** | **0.13** | **-3.73** | **-0.32** | **0.02** |  | **-2.19** | **0.11** | **-3.97** | **-0.42** | **0.02** |
| Relocation |  |  |  |  |  |  | -0.55 | 0.58 | -1.31 | 0.22 | 0.16 |
| GLUM*Relocation |  |  |  |  |  |  | 0.98 | 2.65 | -1.44 | 3.39 | 0.43 |
| **Within effect** | -0.18 | 0.84 | -4.02 | 3.66 | 0.93 |  | -3.10 | 0.05 | -21.38 | 15.18 | 0.74 |
| Relocation |  |  |  |  |  |  | -0.55 | 0.58 | -1.31 | 0.22 | 0.16 |
| GLUM*Relocation |  |  |  |  |  |  | 3.01 | 20.32 | -15.35 | 21.37 | 0.75 |
| Log-transformed coefficients are displayed. PA is given in MET-minutes per week. Between- and within-effects refer to the between- and within-component of the exposure measure and were assessed within the same model. We ran separate models for each exposure measure, i.e. TCD = Tree Cover Density; SAVI = Soil-Adjusted Vegetation Index; GLUM = Green Land Use Mix. Significant results are displayed **in bold.**  **Model 1** adjusted for: sex, lifestyle preference, age, education, marital status, housing price, nonresidential and residential building density, date of questionnaire completion, relocation status. **Model 2** adjusted for: sex, lifestyle preference, age, education, marital status, housing price, nonresidential and residential building density, date of questionnaire completion, relocation status and interaction term with relocation status. | | | | | | | | | | | |

| Table S8. Results sensitivity analyses for greenness exposure and SB including interactions with relocation status (1000m buffer). | | | | | | | | | |
| --- | --- | --- | --- | --- | --- | --- | --- | --- | --- |
|  | Model 1 | | | |  | Model 2 (relocation) | | | |
|  | β | 95% CI | | P |  | β | 95% CI | | P |
| **Sedentary Behavior** | | | | | | | | | |
| **TCD** |  |  |  |  |  |  |  |  |  |
| **Between effect** | -0.43 | -125.50 | 124.64 | 0.99 |  | 32.68 | -105.74 | 171.11 | 0.64 |
| Relocation |  |  |  |  |  | 11.78 | -41.70 | 65.25 | 0.67 |
| TCD*Relocation |  |  |  |  |  | -163.50 | -461.16 | 134.15 | 0.28 |
| **Within effect** | -50.11 | -270.13 | 169.91 | 0.65 |  | -224.02 | -695.17 | 247.14 | 0.35 |
| Relocation |  |  |  |  |  | 11.78 | -41.70 | 65.25 | 0.67 |
| TCD*Relocation |  |  |  |  |  | 209.60 | -294.57 | 713.77 | 0.41 |
| **SAVI** |  |  |  |  |  |  |  |  |  |
| **Between effect** | 205.15 | -214.56 | 624.85 | 0.34 |  | 136.52 | -290.71 | 563.76 | 0.53 |
| Relocation |  |  |  |  |  | -135.18 | -291.84 | 21.47 | 0.09 |
| SAVI*Relocation |  |  |  |  |  | 320.11 | -79.97 | 720.20 | 0.12 |
| **Within effect** | 10.82 | -348.77 | 370.41 | 0.95 |  | -74.45 | -498.73 | 349.82 | 0.73 |
| Relocation |  |  |  |  |  | -135.18 | -291.84 | 21.47 | 0.09 |
| SAVI*Relocation |  |  |  |  |  | 195.51 | -345.24 | 736.26 | 0.48 |
| **GLUM** |  |  |  |  |  |  |  |  |  |
| **Between effect** | 183.14 | -29.08 | 395.37 | 0.09 |  | 156.14 | -60.71 | 373.00 | 0.16 |
| Relocation |  |  |  |  |  | -59.10 | -152.23 | 34.03 | 0.21 |
| GLUM*Relocation |  |  |  |  |  | 157.38 | -133.22 | 447.98 | 0.29 |
| **Within effect** | -6.65 | -406.19 | 392.89 | 0.97 |  | -227.43 | -1978.29 | 1523.43 | 0.80 |
| Relocation |  |  |  |  |  | -59.10 | -152.23 | 34.03 | 0.21 |
| GLUM*Relocation |  |  |  |  |  | 228.46 | -1524.29 | 1981.21 | 0.80 |
| SB is given as sitting time in minutes per day. Between- and within-effects refer to the between- and within-component of the exposure measure and were assessed within the same model. We ran separate models for each exposure measure, i.e. TCD = Tree Cover Density; SAVI = Soil-Adjusted Vegetation Index; GLUM = Green Land Use Mix. Significant results are displayed **in bold.**  **Model 1** adjusted for: sex, lifestyle preference, age, education, marital status, housing price, nonresidential and residential building density, date of questionnaire completion, relocation status. **Model 2** adjusted for: sex, lifestyle preference, age, education, marital status, housing price, nonresidential and residential building density, date of questionnaire completion, relocation status and interaction term with relocation status. | | | | | | | | | |

| Table S9. Results sensitivity analyses for greenness exposure and PA including interactions with relocation status (500m, 800m buffer). | | | | | | | | | | | | | | | | | | | | | | | |
| --- | --- | --- | --- | --- | --- | --- | --- | --- | --- | --- | --- | --- | --- | --- | --- | --- | --- | --- | --- | --- | --- | --- | --- |
|  | **500 meter** | | | | | | | | | | |  | **800 meter** | | | | | | | | | | |
|  | **Model 1** | | | | |  | **Model 2** | | | | |  | **Model 1** | | | | |  | **Model 2** | | | | |
|  | β | exp(β) | 95% CI | | p |  | β | exp(β) | 95% CI | | p |  | β | exp(β) | 95% CI | | p |  | β | exp(β) | 95% CI | | p |
| **Physical Activity** | | | | | | | | | | | | | | | | | | | | | | | |
| **TCD** |  |  |  |  |  |  |  |  |  |  |  |  |  |  |  |  |  |  |  |  |  |  |  |
| **Between effect** | -0.72 | 0.49 | -1.89 | 0.46 | 0.23 |  | -0.18 | 0.84 | -1.51 | 1.16 | 0.80 |  | -0.44 | 0.64 | -1.51 | 0.62 | 0.41 |  | -0.16 | 0.85 | -1.38 | 1.06 | 0.80 |
| Relocation |  |  |  |  |  |  | 0.08 | 1.09 | -0.30 | 0.47 | 0.67 |  |  |  |  |  |  |  | -0.03 | 0.97 | -0.44 | 0.37 | 0.88 |
| TCD*Relocation |  |  |  |  |  |  | **-2.48** | **0.08** | **-5.10** | **0.15** | **0.06** |  |  |  |  |  |  |  | -1.41 | 0.24 | -3.96 | 1.14 | 0.28 |
| **Within effect** | **-2.06** | **0.13** | **-4.06** | **-0.06** | **0.04** |  | -3.61 | 0.03 | -7.47 | 0.26 | 0.07 |  | **-2.25** | **0.11** | **-4.38** | **-0.11** | **0.04** |  | -3.12 | 0.04 | -7.73 | 1.48 | 0.18 |
| Relocation |  |  |  |  |  |  | 0.08 | 1.09 | -0.30 | 0.47 | 0.67 |  |  |  |  |  |  |  | -0.03 | 0.97 | -0.44 | 0.37 | 0.88 |
| TCD*Relocation |  |  |  |  |  |  | 2.03 | 7.62 | -2.22 | 6.28 | 0.35 |  |  |  |  |  |  |  | 1.06 | 2.87 | -3.90 | 6.01 | 0.68 |
| **SAVI** |  |  |  |  |  |  |  |  |  |  |  |  |  |  |  |  |  |  |  |  |  |  |  |
| **Between effect** | -1.69 | 0.19 | -4.67 | 1.30 | 0.27 |  | -1.49 | 0.22 | -4.57 | 1.58 | 0.34 |  | -1.40 | 0.25 | -4.66 | 1.87 | 0.40 |  | -1.41 | 0.24 | -4.76 | 1.94 | 0.41 |
| Relocation |  |  |  |  |  |  | 0.08 | 1.08 | -1.11 | 1.27 | 0.90 |  |  |  |  |  |  |  | -0.24 | 0.78 | -1.50 | 1.01 | 0.70 |
| SAVI*Relocation |  |  |  |  |  |  | -0.86 | 0.42 | -4.08 | 2.35 | 0.60 |  |  |  |  |  |  |  | 0.04 | 1.04 | -3.24 | 3.32 | 0.98 |
| **Within effect** | -0.65 | 0.52 | -4.12 | 2.81 | 0.71 |  | -0.94 | 0.39 | -5.21 | 3.34 | 0.67 |  | -0.16 | 0.85 | -3.81 | 3.49 | 0.93 |  | 0.09 | 1.09 | -4.25 | 4.43 | 0.97 |
| Relocation |  |  |  |  |  |  | 0.08 | 1.08 | -1.11 | 1.27 | 0.90 |  |  |  |  |  |  |  | -0.24 | 0.78 | -1.50 | 1.01 | 0.70 |
| SAVI*Relocation |  |  |  |  |  |  | 0.59 | 1.80 | -4.80 | 5.97 | 0.83 |  |  |  |  |  |  |  | -0.56 | 0.57 | -5.99 | 4.86 | 0.84 |
| **GLUM** |  |  |  |  |  |  |  |  |  |  |  |  |  |  |  |  |  |  |  |  |  |  |  |
| **Between effects** | -1.64 | 0.19 | -3.37 | 0.09 | 0.06 |  | -1.63 | 0.20 | -3.42 | 0.17 | 0.08 |  | -1.79 | 0.17 | -3.48 | -0.11 | 0.04 |  | **-1.92** | 0.15 | **-3.68** | **-0.16** | **0.03** |
| Relocation |  |  |  |  |  |  | -0.21 | 0.81 | -0.84 | 0.41 | 0.50 |  |  |  |  |  |  |  | -0.44 | 0.64 | -1.14 | 0.26 | 0.21 |
| GLUM*Relocation |  |  |  |  |  |  | -0.09 | 0.91 | -2.69 | 2.51 | 0.95 |  |  |  |  |  |  |  | 0.72 | 2.05 | -1.69 | 3.13 | 0.56 |
| **Within effect** | -2.17 | 0.11 | -5.83 | 1.48 | 0.24 |  | -2.75 | 0.06 | -17.76 | 12.27 | 0.72 |  | 0.08 | 1.08 | -3.69 | 3.85 | 0.97 |  | -4.87 | 0.01 | -22.37 | 12.63 | 0.58 |
| Relocation |  |  |  |  |  |  | -0.21 | 0.81 | -0.84 | 0.41 | 0.50 |  |  |  |  |  |  |  | -0.44 | 0.64 | -1.14 | 0.26 | 0.21 |
| GLUM*Relocation |  |  |  |  |  |  | 0.60 | 1.82 | -14.54 | 15.74 | 0.94 |  |  |  |  |  |  |  | 5.13 | 169.00 | -12.37 | 22.63 | 0.57 |
| Log-transformed coefficients are displayed. PA is given in MET-minutes per week. Between- and within-effects refer to the between- and within-component of the exposure measure and were assessed within the same model. We ran separate models for each exposure measure, i.e. TCD = Tree Cover Density; SAVI = Soil-Adjusted Vegetation Index; GLUM = Green Land Use Mix. Significant results are displayed **in bold. Model 1** adjusted for: sex, lifestyle preference, age, education, marital status, housing price, nonresidential and residential building density, date of questionnaire completion, relocation status. **Model 2** adjusted for: sex, lifestyle preference, age, education, marital status, housing price, nonresidential and residential building density, date of questionnaire completion, relocation status and interaction term with relocation status. | | | | | | | | | | | | | | | | | | | | | | | |

| Table S10. Results sensitivity analyses for greenness exposure and SB including interactions with relocation status (500m, 800m buffer). | | | | | | | | | | | | | | | | | | | |
| --- | --- | --- | --- | --- | --- | --- | --- | --- | --- | --- | --- | --- | --- | --- | --- | --- | --- | --- | --- |
|  | **500 meter** | | | | | | | | |  | **800 meter** | | | | | | | | |
|  | **Model 1** | | | |  | **Model 2** | | | |  | **Model 1** | | | |  | **Model 2** | | | |
|  | β | 95% CI | | p |  | β | 95% CI | | p |  | β | 95% CI | | p |  | β | 95% CI | | p |
| **Sedentary Behavior** | | | | | | | | | | | | | | | | | | | |
| **TCD** |  |  |  |  |  |  |  |  |  |  |  |  |  |  |  |  |  |  |  |
| **Between effect** | 34.24 | -100.36 | 168.84 | 0.62 |  | 25.46 | -123.35 | 174.27 | 0.74 |  | 5.19 | -121.21 | 131.59 | 0.94 |  | 21.95 | -117.34 | 161.24 | 0.76 |
| Relocation |  |  |  |  |  | -18.76 | -66.60 | 29.08 | 0.44 |  |  |  |  |  |  | -1.88 | -52.72 | 48.96 | 0.94 |
| TCD*Relocation |  |  |  |  |  | 40.71 | -263.51 | 344.93 | 0.79 |  |  |  |  |  |  | -84.76 | -385.62 | 216.10 | 0.58 |
| **Within effect** | -2.34 | -199.56 | 194.88 | 0.98 |  | -92.76 | -455.16 | 269.64 | 0.62 |  | -32.96 | -248.42 | 182.51 | 0.76 |  | -202.60 | -636.20 | 231.00 | 0.36 |
| Relocation |  |  |  |  |  | -18.76 | -66.60 | 29.08 | 0.44 |  |  |  |  |  |  | -1.88 | -52.72 | 48.96 | 0.94 |
| TCD*Relocation |  |  |  |  |  | 119.36 | -282.96 | 521.68 | 0.56 |  |  |  |  |  |  | 210.44 | -259.20 | 680.08 | 0.38 |
| **SAVI** |  |  |  |  |  |  |  |  |  |  |  |  |  |  |  |  |  |  |  |
| **Between effect** | 78.68 | -281.79 | 439.15 | 0.67 |  | -8.85 | -377.85 | 360.15 | 0.96 |  | 187.86 | -216.65 | 592.37 | 0.36 |  | 104.70 | -307.42 | 516.81 | 0.62 |
| Relocation |  |  |  |  |  | **-157.08** | **-300.84** | **-13.32** | **0.03** |  |  |  |  |  |  | **-155.54** | **-306.25** | **-4.82** | **0.04** |
| SAVI*Relocation |  |  |  |  |  | **395.51** | **10.21** | **780.82** | **0.04** |  |  |  |  |  |  | **379.60** | **-12.86** | **772.06** | **0.06** |
| **Within effect** | 38.72 | -304.69 | 382.13 | 0.82 |  | -14.23 | -424.95 | 396.50 | 0.95 |  | 7.46 | -340.97 | 355.89 | 0.97 |  | -69.04 | -481.79 | 343.70 | 0.74 |
| Relocation |  |  |  |  |  | **-157.08** | **-300.84** | **-13.32** | **0.03** |  |  |  |  |  |  | **-155.54** | **-306.25** | **-4.82** | **0.04** |
| SAVI*Relocation |  |  |  |  |  | 115.70 | -411.87 | 643.27 | 0.67 |  |  |  |  |  |  | 170.58 | -362.08 | 703.24 | 0.53 |
| **GLUM** |  |  |  |  |  |  |  |  |  |  |  |  |  |  |  |  |  |  |  |
| **Between effects** | 20.74 | -181.47 | 222.94 | 0.84 |  | -21.27 | -227.02 | 184.48 | 0.84 |  | 138.30 | -67.82 | 344.43 | 0.19 |  | 101.90 | -108.52 | 312.33 | 0.34 |
| Relocation |  |  |  |  |  | **-78.03** | **-154.17** | **-1.89** | **0.04** |  |  |  |  |  |  | **-71.65** | **-156.97** | **13.68** | **0.10** |
| GLUM*Relocation |  |  |  |  |  | **286.72** | **-23.31** | **596.76** | **0.07** |  |  |  |  |  |  | 215.73 | -75.32 | 506.78 | 0.15 |
| **Within effect** | 139.48 | -224.00 | 502.97 | 0.45 |  | -425.80 | -2068.50 | 1216.89 | 0.61 |  | 19.00 | -359.26 | 397.26 | 0.92 |  | -304.15 | -2066.16 | 1457.87 | 0.73 |
| Relocation |  |  |  |  |  | **-78.03** | **-154.17** | **-1.89** | **0.04** |  |  |  |  |  |  | -71.65 | -156.97 | 13.68 | 0.10 |
| GLUM*Relocation |  |  |  |  |  | 592.23 | -1052.31 | 2236.77 | 0.48 |  |  |  |  |  |  | 335.02 | -1427.44 | 2097.48 | 0.71 |
| SB is given as sitting time in minutes per day. Between- and within-effects refer to the between- and within-component of the exposure measure and were assessed within the same model. We ran separate models for each exposure measure, i.e. TCD = Tree Cover Density; SAVI = Soil-Adjusted Vegetation Index; GLUM = Green Land Use Mix. Significant results are displayed **in bold.**  **Model 1** adjusted for: sex, lifestyle preference, age, education, marital status, housing price, nonresidential and residential building density, date of questionnaire completion, relocation status. **Model 2** adjusted for: sex, lifestyle preference, age, education, marital status, housing price, nonresidential and residential building density, date of questionnaire completion, relocation status and interaction term with relocation status. | | | | | | | | | | | | | | | | | | | |

**Figure S1**. Predicted PA and SB values by sex in the 800m buffer


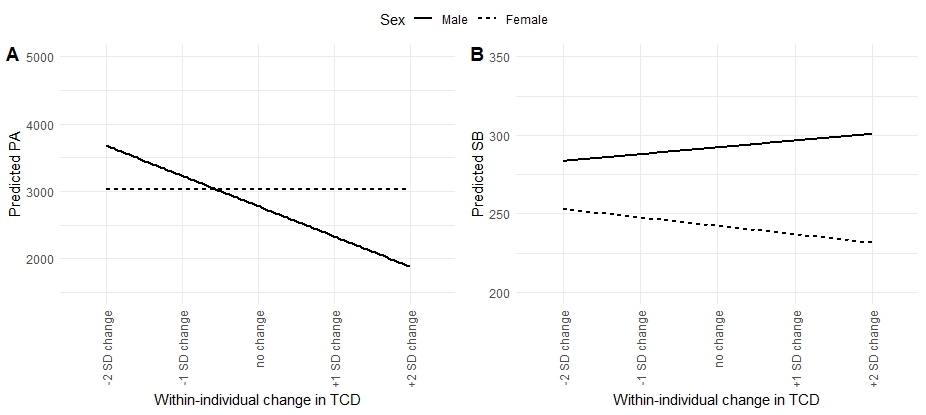


PA = physical activity; TCD = tree cover density

**Figure S2**. Predicted SB values by neighborhood socio-economic status in the 500m buffer

**
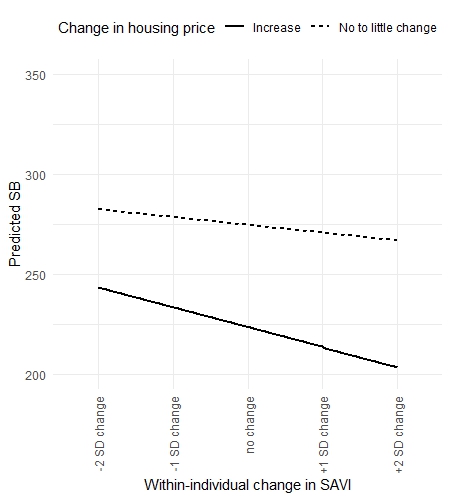
**

SB = sedentary behavior; SAVI = soil-adjusted vegetation index

**Figure S3.** Predicted PA and SB values by lifestyle preference [for being active] in the 500m buffer

**
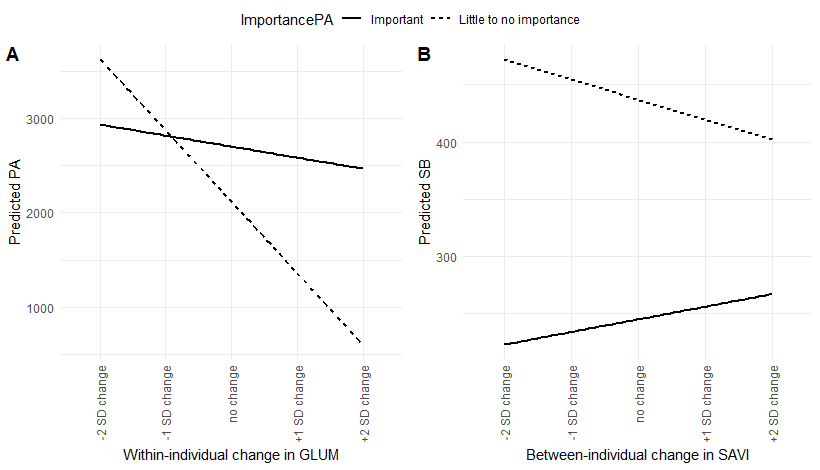
**

PA = physical activity; SB = sedentary behavior; SAVI = soil-adjusted vegetation index; GLUM = green land use mix

**Figure S4.** Predicted SB values by lifestyle preference [for being active] in the 800m buffer


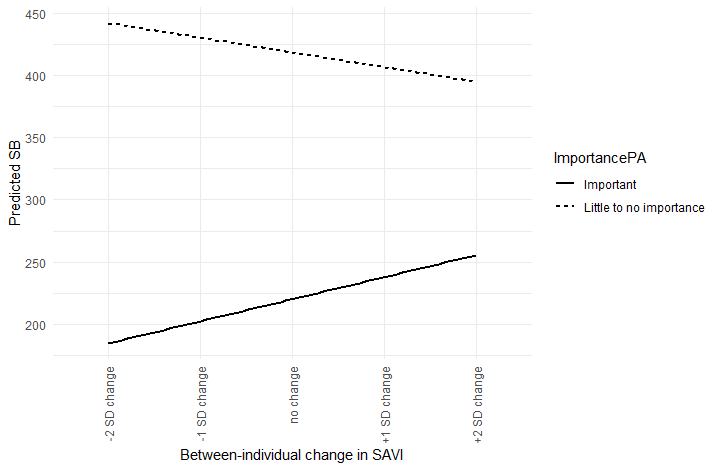


SB = sedentary behavior; SAVI = soil-adjusted vegetation index

**Figure S5**. Predicted PA and SB values by relocation status in the 500m buffer

**
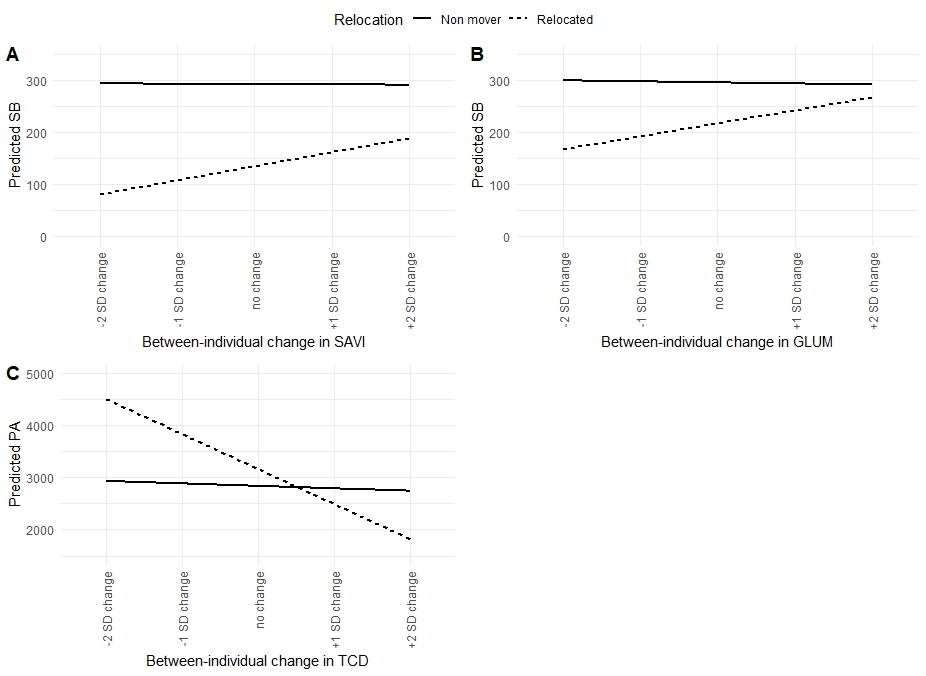
**

PA = physical activity; SB = sedentary behavior; TCD = tree cover density; SAVI = soil-adjusted vegetation index; GLUM = green land use mix

**Figure S6**. Predicted SB values by relocation status in the 800m buffer**
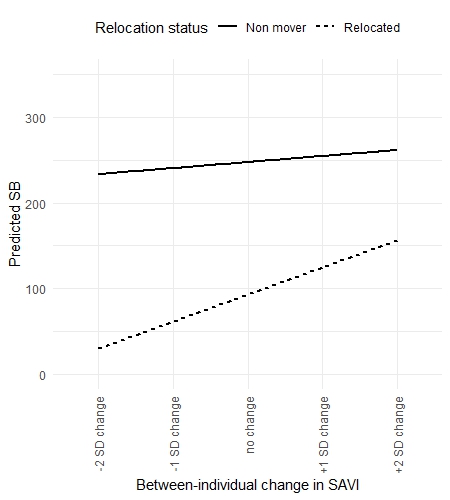
**

SB = sedentary behavior; SAVI = soil-adjusted vegetation index
